# Context-dependent modulation of aggressiveness of pediatric tumors by individual oncogenic RAS isoforms

**DOI:** 10.1038/s41388-021-01904-4

**Published:** 2021-06-25

**Authors:** Julia Bauer, Nicole Cuvelier, Nada Ragab, Katja Simon-Keller, Frauke Nitzki, Natalie Geyer, Dominik S. Botermann, Dominik P. Elmer, Albert Rosenberger, Thomas A. Rando, Stefano Biressi, James A. Fagin, Dieter Saur, Christian Dullin, Hans-Ulrich Schildhaus, Walter Schulz-Schaeffer, Fritz Aberger, Anja Uhmann, Heidi Hahn

**Affiliations:** 1 Department of Human Genetics, University Medical Center Goettingen, Goettingen, Germany; 2 Institute of Pathology, University Medical Center Mannheim, University of Heidelberg, Mannheim, Germany; 3 Department of Biosciences, Paris-Lodron University of Salzburg, Cancer Cluster Salzburg, Salzburg, Austria; 4 Department of Genetic Epidemiology, University Medical Center Goettingen, Goettingen, Germany; 5 Paul F. Glenn Center for the Biology of Aging and Department of Neurology and Neurological Sciences, Stanford University School of Medicine, Stanford, CA USA; 6 Neurology Service, Veterans Affairs Palo Alto Health Care System, Palo Alto, CA USA; 7 Department of Cellular, Computational and Integrative Biology (CIBIO) and Dulbecco Telethon Institute, University of Trento, Povo-Trento, Italy; 8 Memorial Sloan Kettering Cancer Center, New York, NY USA; 9 Klinik und Poliklinik für Innere Medizin II, Klinikum rechts der Isar der TUM, Muenchen, Germany; 10 Institute for Diagnostic and Interventional Radiology, University Medical Center Goettingen, Goettingen, Germany; 11 Institute of Pathology, University Medical Center Essen, Essen, Germany; 12 Department of Neuropathology, Saarland University Medical Center, Homburg, Germany

**Keywords:** Paediatric cancer, Cancer genetics

## Abstract

A prototypic pediatric cancer that frequently shows activation of RAS signaling is embryonal rhabdomyosarcoma (ERMS). ERMS also show aberrant Hedgehog (HH)/GLI signaling activity and can be driven by germline mutations in this pathway. We show, that in ERMS cell lines derived from sporadic tumors i.e. from tumors not caused by an inherited genetic variant, HH/GLI signaling plays a subordinate role, because oncogenic mutations in *HRAS*, *KRAS*, or *NRAS* (collectively named oncRAS) inhibit the main HH target GLI1 via the MEK/ERK-axis, but simultaneously increase proliferation and tumorigenicity. oncRAS also modulate expression of stem cell markers in an isoform- and context-dependent manner. In *Hh*-driven murine ERMS that are caused by a *Patched* mutation, oncHRAS and mainly oncKRAS accelerate tumor development, whereas oncNRAS induces a more differentiated phenotype. These features occur when the oncRAS mutations are induced at the ERMS precursor stage, but not when induced in already established tumors. Moreover, in contrast to what is seen in human cell lines, oncRAS mutations do not alter Hh signaling activity and marginally affect expression of stem cell markers. Together, all three oncRAS mutations seem to be advantageous for ERMS cell lines despite inhibition of HH signaling and isoform-specific modulation of stem cell markers. In contrast, oncRAS mutations do not inhibit Hh-signaling in *Hh-driven* ERMS. In this model, oncRAS mutations seem to be advantageous for specific ERMS populations that occur within a specific time window during ERMS development. In addition, this window may be different for individual oncRAS isoforms, at least in the mouse.

## Introduction

Rhabdomyosarcoma (RMS) is the most common type of soft tissue sarcoma in children with poor prognosis [1]. The major pediatric form is embryonal RMS (ERMS), which accounts for ~75% of RMS (reviewed by e.g. [2]). ERMS originate from muscle progenitor or stem cells [3–5] and contain cell populations with tumor-propagating or cancer stem cell (CSC) features (for review see ref. [6]) that may explain their intratumoral heterogeneity [7, 8].

ERMS is a prototypic RAS-associated pediatric cancer. Indeed, individual oncogenic RAS (oncRAS) mutations affecting all three *RAS* genes (*HRAS*, *KRAS,* and *NRAS*) occur in up to 42% of ERMS [9–11]. The current discussion hinges on whether oncRAS mutations are ERMS drivers or rather modifiers. In favor of the “driver-hypothesis” are studies in zebrafish [12] and genomic analyses including whole genome sequencing analysis [7, 10, 11]. Moreover, patients with Noonan or Costello syndrome, which are caused by activating *K-, N*- or *HRAS* germline mutations, respectively, are predisposed to ERMS [13]. On the other hand, microarray-based data showing that a RAS signature exists together only with signatures from other activated signaling pathways [5] and the fact that oncRAS mutations are found in ERMS only in combination with other mutations and do not lead to ERMS when occuring alone in the mouse [14–17] favor the “modifier-hypothesis”. Altogether oncRAS mutations seem to play a very important role in ERMS pathogenesis although their exact role still remains to be clarified.

Another pathway that is active in ERMS is Hedgehog (HH) signaling [18–20]. The major players of HH signaling are the HH ligands, the HH receptor Patched1 (PTCH), the PTCH interaction partner Smoothened (SMO) and GLI transcription factors. Aberrant activation of HH signaling leads to a variety of tumors including ERMS (reviewed by e.g., ref. [21]). Indeed, inherited *PTCH/Ptch* mutations can result in ERMS formation both in humans and mice [22, 23]. The most reliable read-out of the pathway’s activity is the transcriptional level of *GLI1* (reviewed by e.g., refs. [24, 25]. We and others found that the expression of *GLI1* and other HH targets is higher in ERMS compared to alveolar RMS (ARMS) [18, 19]. However, sporadic ERMS lack canonical HH signaling activity via the HH/PTCH/SMO/GLI axis [26, 27] and GLI activity is apparently regulated in a non-canonical manner, which summarizes the regulation of GLI transcription factors by interaction with other signaling pathways including the RAS pathway (reviewed by e.g., refs. [24, 28].

Here we compared the influence of oncH-, oncK-, and oncNRAS mutations on ERMS growth in different experimental settings using human ERMS cell lines derived from sporadic ERMS and the *Ptch*^*+/−*^ mouse model that develops ERMS-like tumors due to inherited *Ptch* mutations [23]. This allowed us to investigate the impact of oncRAS mutations on early and late ERMS stages and also on canonical or non-canonical HH/GLI-signaling in ERMS.

## Results

### OncRAS mutations can inhibit GLI1/*GLI1* expression via the MEK/ERK axis in human ERMS cell lines

**Fig. 1:**
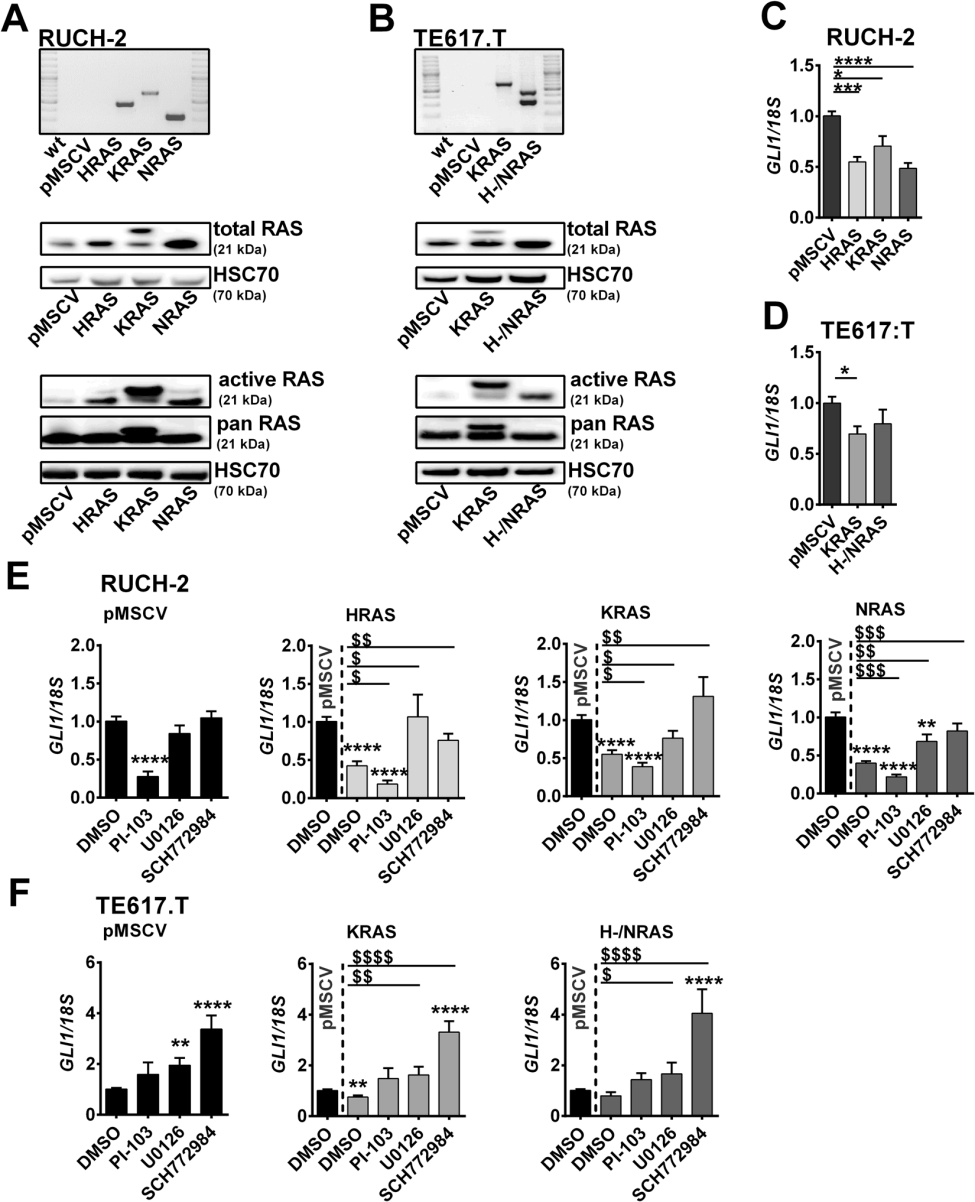
Impact of oncRAS mutations on *GLI1* expression in RUCH-2 and TE617.T ERMS cells. **A**, **B** Expression of HRAS, KRAS, NRAS, or pMSCV was confirmed by RT-PCR on cDNA level (top), western blot analyses for RAS protein (middle) and RAS activity by RAS-GTP pulldown assay (*n* = 2, bottom) in (**A**) RUCH-2 and (**B**) TE617.T cell lines. HSC70 was used as loading control. **C**, **D** *GLI1* qRT-PCR analyses of (**C**) HRAS-, KRAS-, NRAS-expressing RUCH-2 and (**D**) KRAS- or H-/NRAS-expressing TE617.T cells compared to respective pMSCV control cells. **E**, **F** *GLI1* qRT-PCR analyses of (**E**) HRAS-, KRAS- or NRAS-expressing RUCH-2 and **F** KRAS- or H-/NRAS-expressing TE617.T cells treated with 3 µM PI-103-, 10 µM U0126- or 0.5 µM SCH772984 compared to pMSCV cells. DMSO-treated (1 µl/ml) cells served as controls. Data are shown as fold induction over the expression level of solvent-treated pMSCV control cells, which was set to 1. Bars show mean + SEM. * or ^$^: significant compared to solvent-treated pMSCV control or solvent-treated oncRAS cell line tested by Mann–Whitney test. *^/$^ *p* < 0.05, **^/$$^ *p* < 0.01, ***^/$$$^ *p* < 0.001, ****^/$$$$^ *p* < 0.0001.

In order to investigate the impact of oncRAS mutations on non-canoncial HH/GLI signaling activity in established human sporadic ERMS, the RAS wildtype ERMS cell lines RUCH-2 and TE617.T were stably transduced with *pMSCVpuro-HRAS*^*G12V*^, *pMSCVpuro-KRAS*^*G12V*^, *pMSCVpuro-NRAS*^*G12V*^, or the *pMSCVpuro* empty vector (HRAS, KRAS, NRAS, or pMSCV, respectively). DNA integration and cDNA expression was demonstrated by PCR and RAS protein expression (Fig. 1A, B; KRAS^G12V^ is HA-tagged and is larger than the endogenous KRAS protein). Unfortunately, HRAS and NRAS were expressed in the same TE617.T cell clone, probably due to inadvertent transduction with both vectors (Fig. 1B). Elevated RAS activity was verified by RAS activation assay (Fig. 1A, B).

Our results show that oncRAS mutations can negatively regulate *GLI1* mRNA expression in RUCH-2 and TE617.T (Fig. 1C, D; results for TE617.T H-/NRAS are not significant). To investigate whether the two main RAS-downstream pathways are involved in suppression of *GLI1*, the cells were incubated with the AKT/mTOR inhibitor PI-103, the MEK inhibitor U0126 or the ERK inhibitor SCH772984 (see Fig. S1A–C for inhibitor functionality). Whereas PI-103 downregulated *GLI1* in both control and oncRAS-transduced RUCH-2 cells (Fig. 1E;), incubation of RUCH-2 oncRAS cells with U0126 or SCH772984 restored *GLI1* expression to basal levels of control cells (Fig. 1E). This was also seen in RD cells (Fig. S1B) that harbor a *NRAS*^*Q61H*^ mutation [29]. In TE617.T cells, PI-103 had no effect and MEK and/or ERK inhibit *GLI1* expression independently of oncRAS, because U0126 and SCH772984 elevated *GLI1* also in control cells (Fig. 1F). This might be due to mutations in *MAP3K14/*NIK or *MAP3K1*/MEKK1 of TE617.T cells, which result in an alternative splice variant or a threonine deletion at position 949, respectively (The Cancer Cell Line Encyclopedia [30]). The function of these changes is unknown. However, because these proteins can activate ERK [31, 32], these changes may have influenced the activity of ERK. Indeed, SCH772984-mediated pERK suppression was weaker compared to RUCH-2 cells (Fig. S1C).

**Fig. 2:**
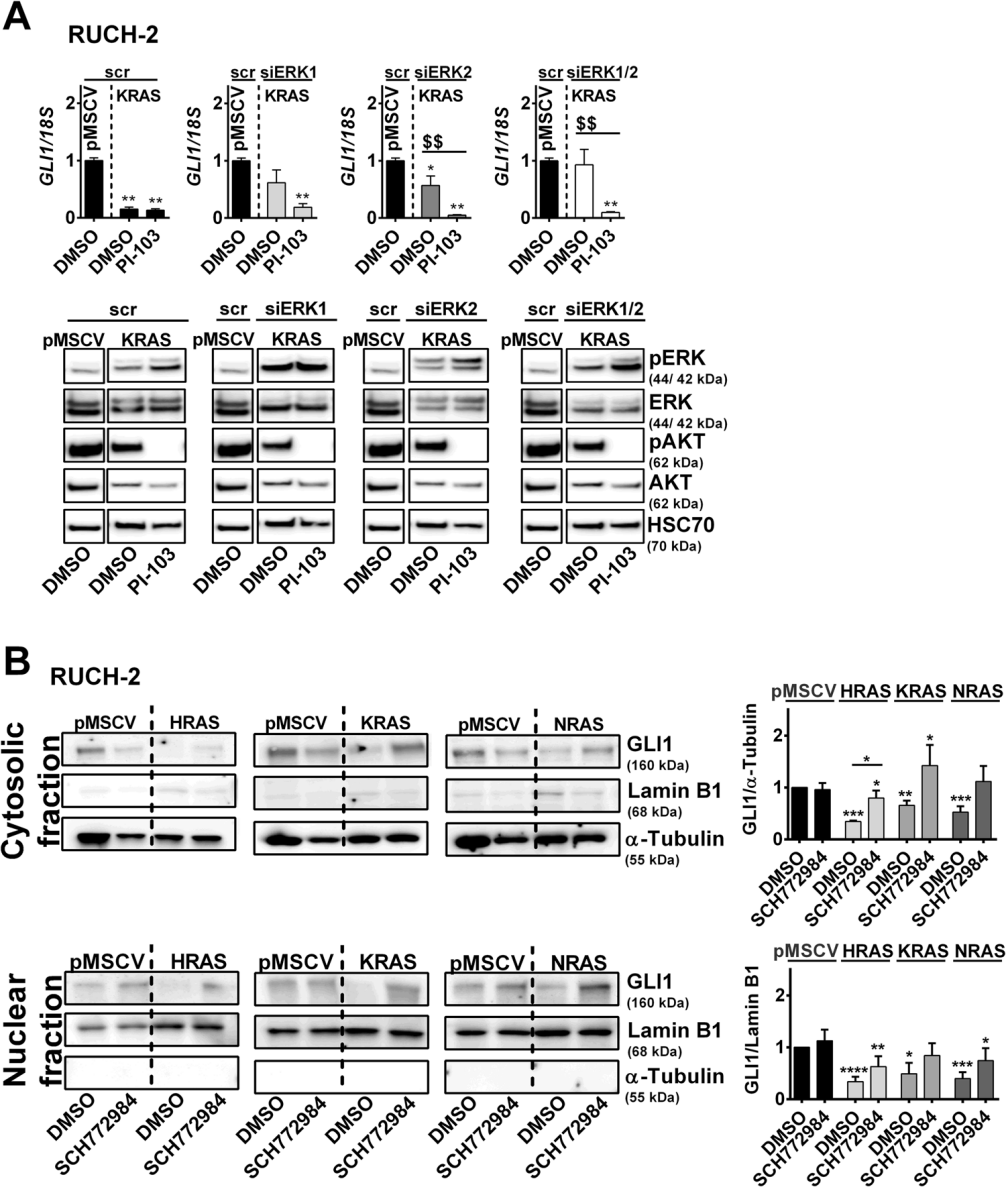
Impact of ERK on *GLI1* expression in RUCH-2 cells. **A** *GLI1* expression (top) and representative pERK/ERK and pAKT/AKT western blot analyses (bottom) (*n* = 2) of KRAS-expressing RUCH-2 cells after siRNA (100 nM each)-mediated ERK1 and/or ERK2 knockdown with and without PI-103 treatment (3 µM) compared to scramble (scr) siRNA transfected KRAS-expressing RUCH-2 and pMSCV control cells. HSC70 served as loading control. **B** Representative western blot analyses (*n* = 5) (left) and respective densitometric analyses (right) of GLI1 expression in cytosolic and nuclear fractions of HRAS-, KRAS- and NRAS-expressing RUCH-2 cells with or without SCH772984 treatment in comparison to solvent-treated pMSCV control. Lamin B1 or α-Tubulin served as loading controls for nuclear or cytosolic fractions, respectively. Bars: mean + SEM. * or ^$^: significant compared to solvent-treated pMSCV control or solvent-treated oncRAS cell line tested by Mann–Whitney test. *^/$^ *p* < 0.05, **^/$$^ *p* < 0.01, ***^/$$$^ *p* < 0.001, ****^/$$$$^ *p* < 0.0001.

To confirm that ERK is involved in *GLI1* suppression, ERK1 and/or ERK2 expression was decreased in RUCH-2 KRAS cells by transient siRNA transfection. Indeed, even a partial ERK1 and/or ERK2 knockdown restores *GLI1* expression to basal levels of pMSCV control cells (Fig. 2A; not completely restored by ERK2 knockdown), indicating that ERK suppresses *GLI1*. The data also show that the ERK knockdown does not influence PI-103-mediated downregulation of *GLI1* (Fig. 2A). This implicates that the AKT axis rather activates *GLI1* expression, at least in RUCH-2 KRAS cells. However, this situation is certainly much more complex, because in dependency of the cell line (i) PI-103-mediated *GLI1* suppression is associated with ERK phosphorylation (Fig. 2A; Fig. S1A–C), (ii) ERK1 and ERK2 can influence phosphorylation of each other (Fig. 2A), and (iii) U0126 and SCH772984 can impact AKT phosphorylation (Fig. S1A–C).

We also analyzed whether oncRAS alters the intracellular distribution of GLI1. However, oncRAS downregulated GLI1 protein regardless of the cellular compartment and SCH772984 upregulated GLI1 protein back to normal levels (Fig. 2B).

Together, oncRAS can suppress *GLI1*/GLI1 expression and thus HH signaling in ERMS, which can involve the MEK/ERK axis.

### Despite attenuation of HH signaling activity, oncRAS mutations can increase proliferation and tumorigenicity of human ERMS cell lines

**Fig. 3:**
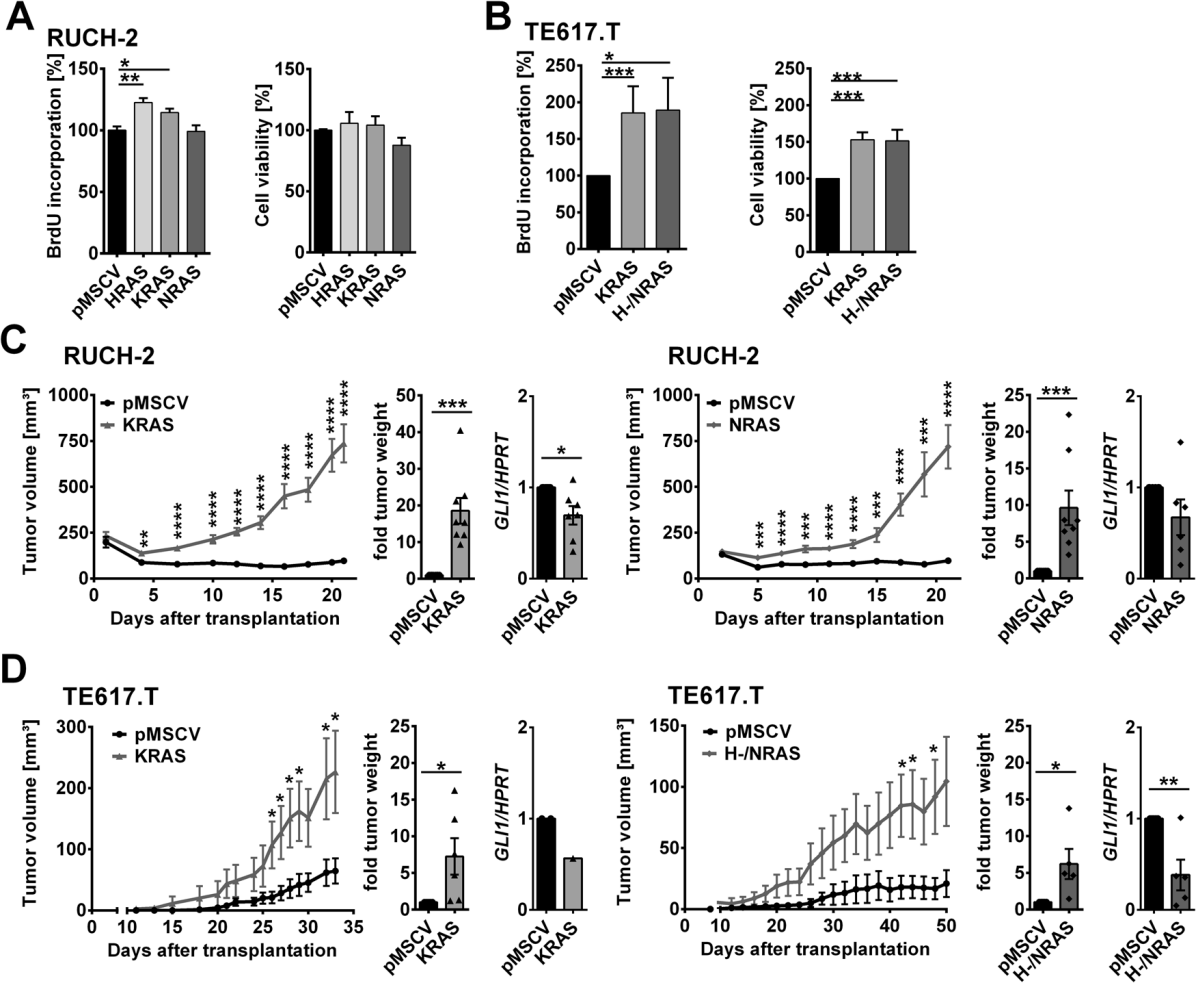
Impact of oncRAS on growth of RUCH-2 and TE617.T cells and on *GLI1* expression after xenotransplantation. **A**, **B** BrdU-incorporation (left) and WST-1 cell viability (right) assays of **A** RUCH-2 (*n* = 3) and **B** TE617.T (*n* = 7) cells stably expressing HRAS, KRAS, NRAS or H-/NRAS. **C**, **D** Mean tumor volume (±SEM), -fold tumor weight and -fold *GLI1* expression of **C** RUCH-2 and **D** TE617.T xenotransplants expressing KRAS (*n* = 8 mice), NRAS (*n* = 8 mice) or H-/NRAS (*n* = 6 mice) compared to respective pMSCV control tumors of the same mice (controls were all set to 1 for -fold tumor weight and -fold *GLI1* expression). Bars: mean + SEM. *Significant by multiple unpaired t-test (tumor growth curve) or Mann–Whitney test (BrdU and WST assay, tumor weight, *GLI1* expression) in comparison to pMSCV controls. *p < 0.05, **p < 0.01, ***p < 0.001, ****p < 0.0001.

Next, we investigated the impact of oncRAS mutations on ERMS growth. BrdU incorporation in a timeframe of 72 h revealed a significant increase in proliferation of oncHRAS- and oncKRAS-, but not of oncNRAS-expressing RUCH-2 cells (Fig. 3A, left panel). Cell viability was not affected (Fig. 3A right panel). In TE617.T cells, proliferation rate and cell viability were significantly increased upon oncKRAS or oncH-/NRAS expression as measured in a timeframe of 24 h by BrdU incorporation and WST-1 assays, respectively (Fig. 3B). Cellular appearance was never affected (Fig. S1D). To evaluate the in vivo growth behavior, control and oncRAS cell lines were transplanted into nude mice. Indeed, all oncRAS cell lines including oncNRAS-expressing RUCH-2 cells showed a significantly accelerated growth and end point weight (Fig. 3C, D). In general, *GLI1* expression remained downregulated compared to the corresponding control xenotransplants. *GLI1*-downregulation was significant for oncKRAS-expressing RUCH-2 and oncH-/NRAS expressing TE617.T xenotransplants (Fig. 3C, D). Although the exact roles of oncH- and oncNRAS in TE617.T remains to be determined, the data demonstrate that oncRAS can accelerate proliferation and tumorigenicity of ERMS cell lines despite downregulation of HH signaling.

### OncRAS mutations can alter the expression of stem cell markers of human ERMS cell lines in an isoform- and context-dependent manner

**Fig. 4:**
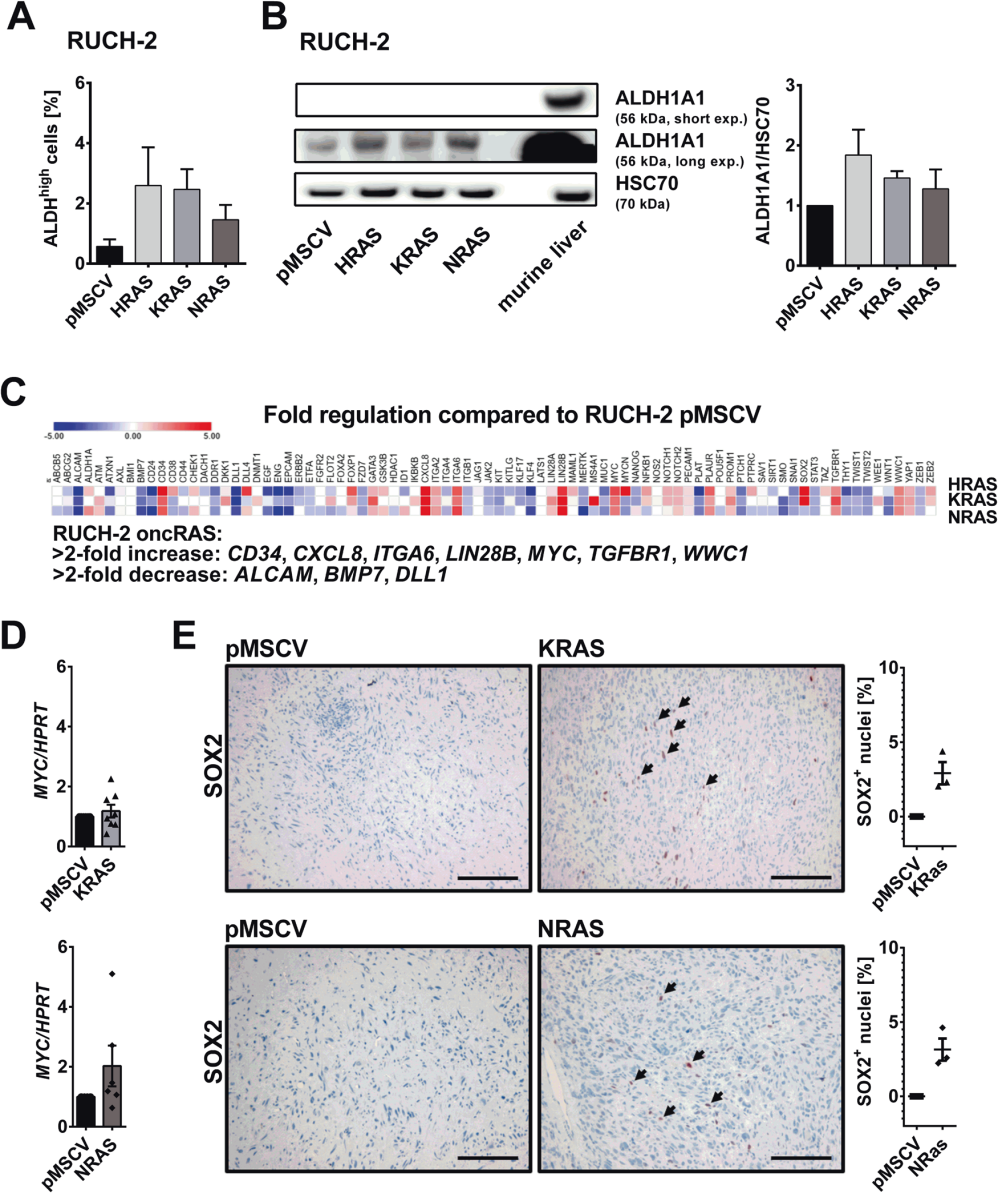
Impact of oncRAS on expression of stem cell markers in RUCH-2 cells. **A** Percentage of Aldefluor^high^ (ALDH^high^) subpopulations of RUCH-2 cells stably expressing HRAS, KRAS, NRAS or pMSCV (*n* = 3) measured by flow cytometry. **B** Representative western blot (*n* = 2) (left) and corresponding densitometric analyses (right) of ALDH1A1 expression in HRAS-, KRAS-, and NRAS-expressing RUCH-2 cells in comparison to RUCH-2 pMSCV control cells. Protein lysate of murine liver served as positive control. **C** Mean fold regulation of 84 cancer stem cell-associated genes in HRAS-, KRAS-, and NRAS-expressing RUCH-2 cells compared to RUCH-2 pMSCV cells (*n* = 2). **D** *MYC* qRT-PCR analyses and **E** anti-SOX2 antibody stainings and percentage of SOX2^+^ nuclei of KRAS and NRAS-expressing RUCH-2 xenotransplants (*n* = 8 or *n* = 3 for *MYC* qRT-PCR or SOX2 stainings, respectively) compared to respective pMSCV control tumors of the same mice (set to 1 for *MYC* qRT-PCR). Bars in **A**, **D**: mean + SEM. Scale bars: 100 µm. Arrows: SOX2^+^ nuclei.

Because oncRAS can change the expression of cancer stem cell (CSC) markers [33], oncRAS-expressing RUCH-2 were analyzed in a pilot experiment for the activity of aldehyde dehydrogenase (ALDH) that is associated with self-renewal and tumor formation capacity in RD cells [34]. Indeed, as revealed by Aldeflour assay, oncRAS-expressing RUCH-2 cells showed a slight, but not significant increase in ALDH^high^ cells compared to control cells (Fig. 4A). This was also seen on protein level (Fig. 4B). Next, we analyzed 84 CSC-associated genes of a commercially available RT-PCR array. Using arbitrary fold change cut-offs of >2 and <2, all oncRAS RUCH-2 cells showed upregulated *CD34*, *CXCL8*, *ITGA6*, *LIN28B*, *MYC*, *TGFBR1*, and *WWC1*, and downregulated *ALCAM*, *BMP7*, and *DLL1* expression (Fig. 4C). All other genes did not meet the cut-off criteria or were differentially regulated by individual oncRAS isoforms like *SOX2*, which was upregulated by oncKRAS and oncHRAS, but not by oncNRAS (Fig. 4C). To investigate whether this expression pattern was retained after transplantation, *MYC* or SOX2 expression of oncKRAS and oncNRAS RUCH-2 xenotransplants was examined by qRT-PCR or immunhistological stainings, respectively. These approaches showed that *MYC* expression was no longer significantly elevated after transplantation (Fig. 4D) and that oncNRAS xenotransplants started to expressed SOX2 (Fig. 4E). The latter observation could explain why oncNRAS expressing RUCH-2 cells robustly grew in vivo (Fig. 3C), but hardly grew in vitro (Fig. 3A, right panel). Together, although the data are very preliminary, the experiments implicate that oncRAS modulate expression of CSC-associated genes in an isoform- and context-dependent manner.

### Without affecting Hh signaling activity, oncHRAS or oncKRAS induction at the ERMS precursor stage in *Ptch*^*+/−*^ mice accelerates tumor growth, whereas oncNRAS results in a more differentiated tumor phenotype

To test the influence of oncRAS mutations on growth and HH signaling activity in Hh-driven ERMS, oncRAS mutations were induced in ERMS of *Ptch*^*+/−*^ mice, which are wildtype for *Hras*, *Kras*, and *Nras* (*oncRas* mutations were excluded by sequencing). This model also allowed us to study the impact of oncRAS mutations on early and late ERMS stages.

Because ERMS of *Ptch*^*+/−*^ mice highly express *Myf5* [35, 36], the *Myf5*^*CreER*^ Cre-driver [37] was used to conditionally activate oncRAS mutations in the tumors. The *Myf5*^*CreER*^ Cre-driver’s activity in ERMS and at the 3 *Ras* loci was confirmed by lineage tracing using *Ptch*^*+/−*^*R26R*^*+/−*^*Myf5*^*CreER/+*^ mice (Fig. S2A) and by specific recombination assays (Fig. S2B; ERMS of *Ptch*^*+/−*^*oncRas*^*fl/+*^*Myf5*^*CreER/wt*^ mice showing spontaneous recombination were excluded from the analyses), respectively. Additionally, tamoxifen-mediated effects on ERMS growth were excluded (Fig. S3A).

Because germline mutations of oncHRAS and oncKRAS or oncNRAS do not result in ERMS or are lethal, respectively [14–17] and because ERMS in *Ptch*^*+/−*^ mice are initiated before birth and become palpable at the earliest around 8 weeks of age [36], oncRAS mutations were induced in 4 weeks old *Ptch*^*+/−*^*oncRas*^*fl/+*^*Myf5*^*CreER/wt*^ mice. This allowed for analysis of oncRAS-associated effects on already initiated ERMS precursor lesions.

**Fig. 5:**
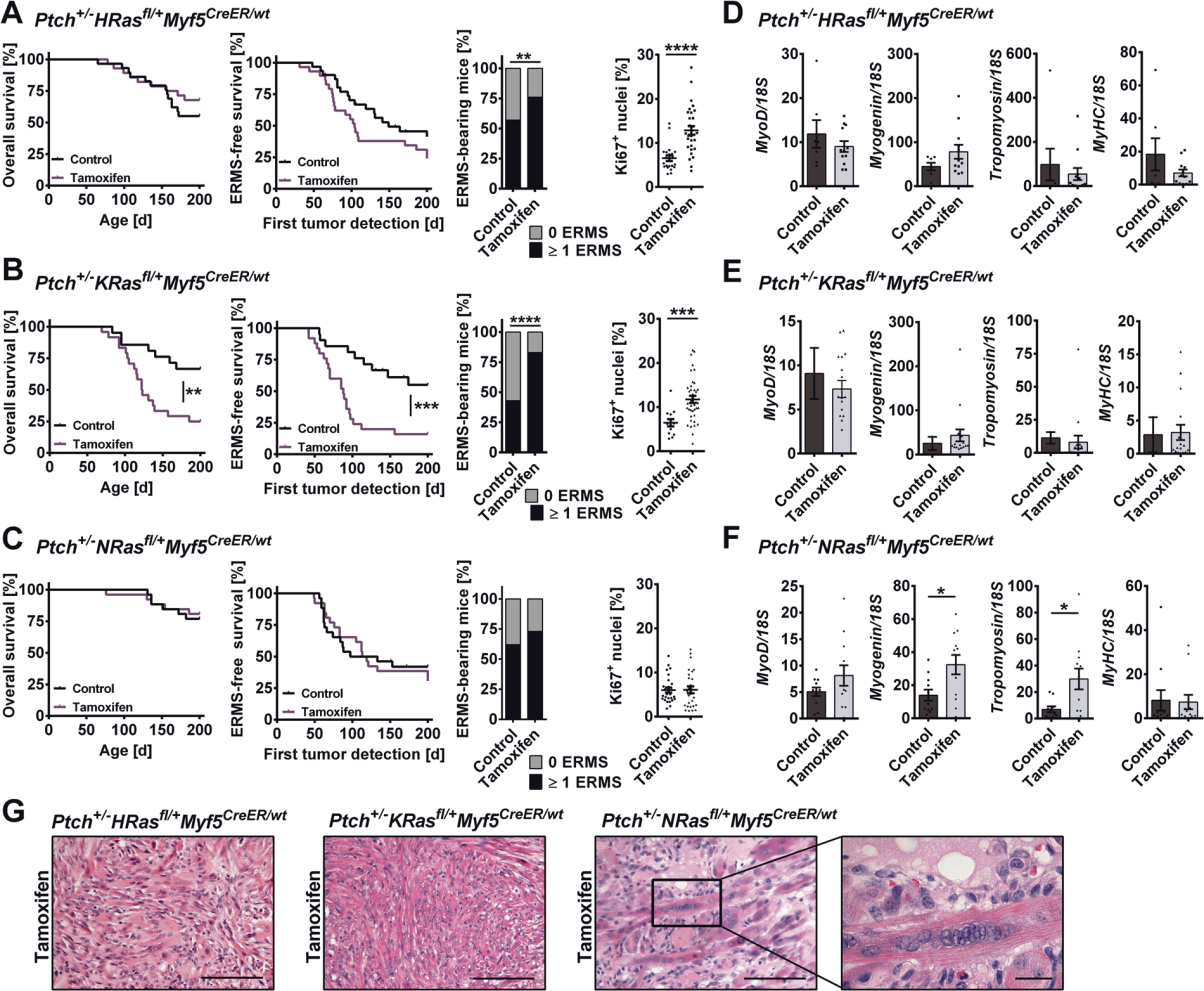
Impact of oncRAS mutations on progression and differentiation of ERMS precursors in *Ptch*^*+/−*^ mice. **A**–**C** ERMS development in **A** *Ptch*^*+/−*^*HRas*^*fl/+*^*Myf5*^*CreER/wt*^, **B** *Ptch*^*+/−*^*KRas*^*fl/+*^*Myf5*^*CreER/wt*^ or **C** *Ptch*^*+/−*^*NRas*^*fl/+*^*Myf5*^*CreER/wt*^ mice injected with tamoxifen at an age of 4 weeks in comparison to the control. Numbers of animals and tumors included in the experiments are given in Table 1. From left to right: overall survival, ERMS-free survival (only palpable ERMS), total ERMS incidence (palpable and non-palpable ERMS) and percentage of Ki67^+^ nuclei in ERMS tissue sections. Ki67 staining was done on 10–22 mice of each cohort. Statistical evaluation was done by Log-rank (Mantel–Cox) testing for Kaplan–Meyer curves and by Chi-square testing for tumor incidence. Dots represent the mean percentage of Ki67^+^ nuclei in individual tumors. **D**–**F** qRT-PCR analyses of *MyoD*, *Myogenin*, *Tropomyosin 3* and *Myosin heavy chain* (MyHC) in ERMS shown as fold expression of the same gene in normal muscle of the same mouse, which was set to 1. **G** Representative H&E stainings of ERMS. Close up: multinucleated cells. Scale bars: 100 µm or 20 µm (close up). For all experiments untreated mice served as controls. Bars: mean ± SEM; dots: individual tumors. *p < 0.05, **p < 0.01, ***p < 0.001, ****p < 0.0001 compared to control *Ptch*^*+/−*^*oncRas*^*fl/+*^*Myf5*^*CreER/wt*^ mice from the respective cohort and tested by non-parametric *t*-tests (Mann–Whitney).

ERMS development was monitored by palpation for 200 days and sacrificed mice were examined for non-palpable tumors. Compared to the controls, ERMS incidence (palpable and non-palpable ERMS) was significantly higher in tamoxifen-treated *Ptch*^*+/−*^*HRas*^*fl/+*^*Myf5*^*CreER/wt*^ and *Ptch*^*+/−*^*KRas*^*fl/+*^*Myf5*^*CreER/wt*^ mice (Fig. 5A, B, Table 1). Although both oncHRAS and oncKRAS enforced tumor proliferation (Fig. 5A, B, right panels), only oncKRAS significantly decreased median overall or ERMS-free survival (only palpable ERMS; Fig. 5B, Table 1) and increased tumor multiplicity (mice with ≥2 ERMS; Table 1). In contrast, oncNRAS did not influence any of these parameters (Fig. 5C, Table 1).

**Table 1:** Mice analyzed for the impact of oncRAS on ERMS precursor lesions.

Treatment	Number of animals	Median overall survival (range)	Healthy // early death	Mice with ERMS (palpable and non-palpable)	Mice with palpable ERMS	Mice with ≥ 2 ERMS (palpable and non-palpable)	Median latency time of palpable ERMS	Further findings (number of animals)
*Ptch*^*+/−*^*HRas*^*fl/+*^*Myf5*^*CreER/wt*^								
Control	30	200 days (65–211)	18 // 12	17 (57 %)	14 (47 %)	6 (20 %)	97 days	*Cysts/Cavernous angioma(4)*, *Medullo-blastoma(3), Papilloma (1)*
Tamoxifen	29	200 days (78–204)	19 // 9	22 (76 %)	19 (66 %)	6 (21 %)	85 days	*Cysts/Cavernous angioma(4), Medullo-blastoma(1), Papilloma (1)*
*Ptch*^*+/****−***^*KRas*^*fl/+*^*Myf5*^*CreER/wt*^								
Control	21	200 days (83–209)	14 // 7	9 (43 %)	8 (38 %)	4 (19 %)	103 days	*Cysts/Cavernous angioma (2), Medullo-blastoma (3)*
Tamoxifen	24	122 days (69–204)	6 // 18	20 (83 %)	17 (58 %)	12 (50 %)	85 days	*Cysts/Cavernous angioma (4), Medullo-blastoma (0)*
*Ptch*^*+/−*^*NRas*^*fl/+*^*Myf5*^*CreER/wt*^								
Control	26	200 days (131–212)	20 // 6	16 (62 %)	14 (54 %)	10 (38 %)	70 days	*Cysts/Cavernous angioma (4), Medullo-blastoma (2)*
Tamoxifen	26	200 days (76–206)	21 // 5	19 (73 %)	17 (65 %)	8 (31 %)	94 days	*Cysts/Cavernous angioma (6), Medullo-blastoma (1)*
*Ptch*^*+/−*^								
Control	24	200 days (60–208)	20 // 4	12 (50 %)	12 50 %)	3 (13 %)	80 days	*Cysts/Cavernous angioma (5), Medullo-blastoma (0)*
Tamoxifen	26	200 days (90–209)	19 // 7	12 (46 %)	12 (46 %)	4 (15 %)	105 days	*Cysts/Cavernous angioma (4), Medullo-blastoma (2)*

The ERMS-like tumors develop mostly at the extremities. They may also develop in muscles of the belly and the back. Intraperitoneal localized tumors were discovered by manual palpation or upon autopsy, as were very small tumors. There were no significant differences in tumor locations between the genotypes. Medulloblastomas harm the animals. These mice were immediately sacrificed and checked for non-palpable ERMS. The coloring of the table is very confusing.

Expression of the Hh downstream targets *Gli1*, *Gli2*, *Ptch1*, and *Hhip* (Fig. S3B–D) and of *MyoD* and *MyHC* (Fig. 5D–F) was not changed. However, in contrast to oncHRAS (Fig. 5D) or oncKRAS (Fig. 5E), oncNRAS significantly increased the expression of the early and late differentiation markers *Myogenin* and *Tropomyosin 3*, respectively (Fig. 5F; see Fig. S3E–G for immunehistochemical analyses of Tropomyosin 3 and MyHC). This went along with the appearance of multinucleated cells (Fig. 5G, right panel), which were rarely observed in the other cohorts (Fig. 5G, middle and left panels).

These data indicate that ERMS precursor lesions of *Ptch*^*+/−*^ mice react differently to the induction of oncRAS isoforms. Thus, oncHRAS and oncKRAS mutations reinforce development of full-blown tumors, with oncKRAS being more aggressive. In contrast, oncNRAS apparently induces a more differentiated phenotype.

### OncRAS mutations do not alter growth of already established ERMS of *Ptch*^*+/−*^ mice

**Fig. 6:**
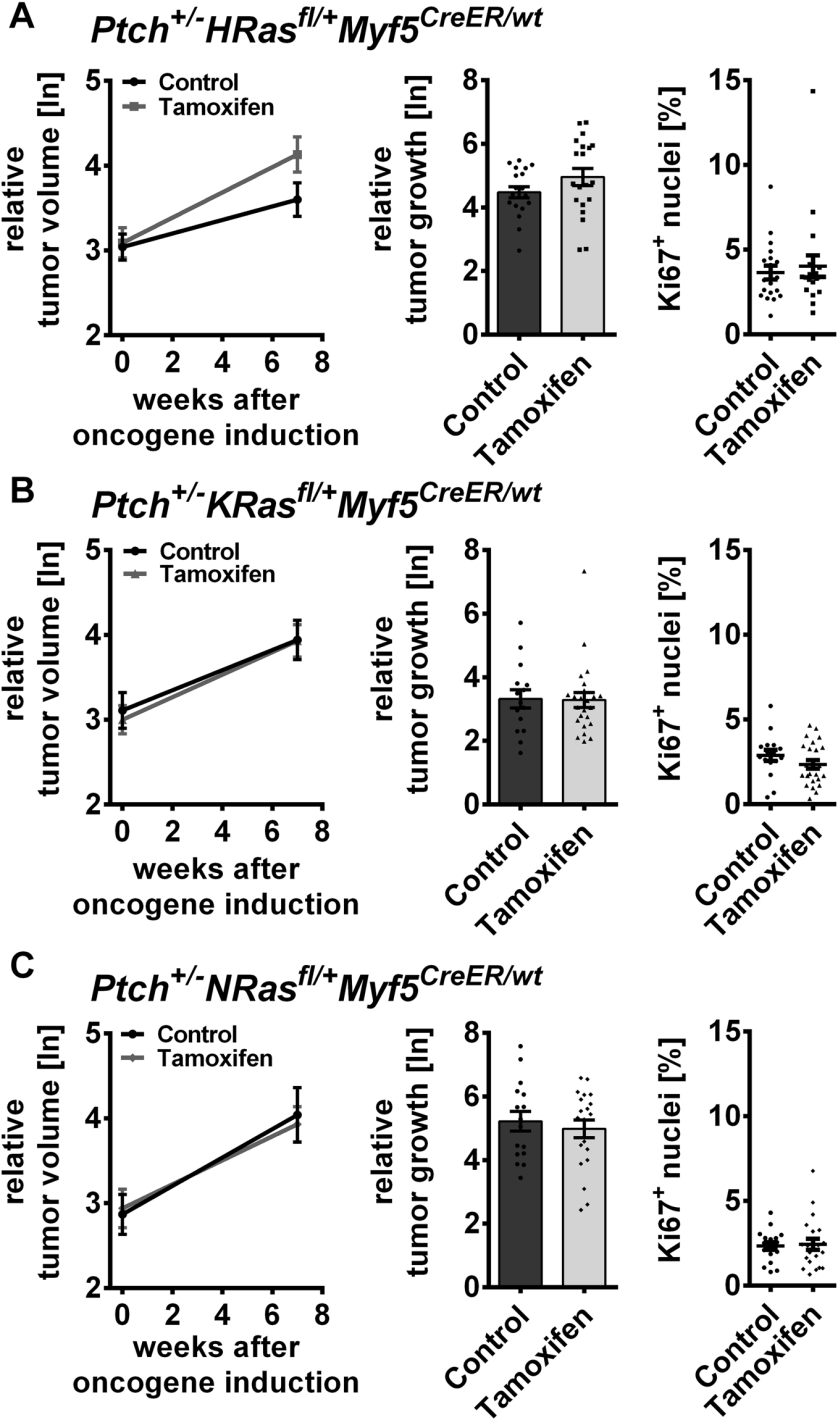
Influence of oncRAS mutations on established ERMS in *Ptch*^*+/−*^ mice. **A**–**C** ERMS growth monitored by µCT measurements before and 7 weeks after tamoxifen-mediated induction of the oncRAS mutations in **A** *Ptch*^*+/−*^*HRas*^*fl/+*^*Myf5*^*CreER/wt*^ **B** *Ptch*^*+/−*^*KRas*^*fl/+*^*Myf5*^*CreER/wt*^ or **C** *Ptch*^*+/−*^*NRas*^*fl/+*^*Myf5*^*CreER/wt*^ mice. At least 12 animals were analyzed per cohort. Left: mean relative tumor volumes before and 7 weeks after injection (ln: logarithmic scale). Middle: relative growth of individual tumors (logarithmic scale). Right: percentage of Ki67^+^ nuclei in the tumors. Solvent-treated mice served as controls. All ERMS of the same mouse were analyzed as individual tumors. Dots: results for individual tumors. Statistical analyses of mean tumor growth or the individual tumor growth and the percentage of Ki67+ nuclei were done by Student’s *t*-tests or non-parametric *t*-tests (Mann–Whitney), respectively.

Next, oncRAS mutations were induced in mice with palpable tumors (~0.5 cm diameter). As measured by μCT the sizes of all ERMS of the *Ptch*^*+/−*^*oncRas*^*fl/+*^*Myf5*^*CreER/wt*^ cohorts were almost identical at onset of the study and after 7 weeks all tumors had grown (Fig. 6A–C, left panels). However, despite efficient Cre-mediated recombination (Fig. S2B) and enhanced intratumoral RAS activity (Fig. S4A), none of the oncRAS isoforms influenced ERMS growth (Fig. 6A–C; left panels). This was confirmed by the relative increase of individual tumor sizes and by Ki67 expression (Fig. 6A–C, middle and right panels). Moreover, the mutations had no impact on the expression of Hh and differentiation markers (Fig. S4B–D). Tamoxifen-mediated effects on tumor growth (Fig. S5 upper panel), expression of *Gli1* and on myogenic differentiation markers (Fig. S5 lower panel) were also excluded.

These results suggest that none of the three oncRAS isoforms influences growth, proliferation or molecular characteristics of full-blown ERMS of *Ptch*^*+/−*^ mice.

### OncRAS mutations do not affect expression of selected stem cell markers in ERMS of *Ptch*^*+/−*^ mice

**Fig. 7:**
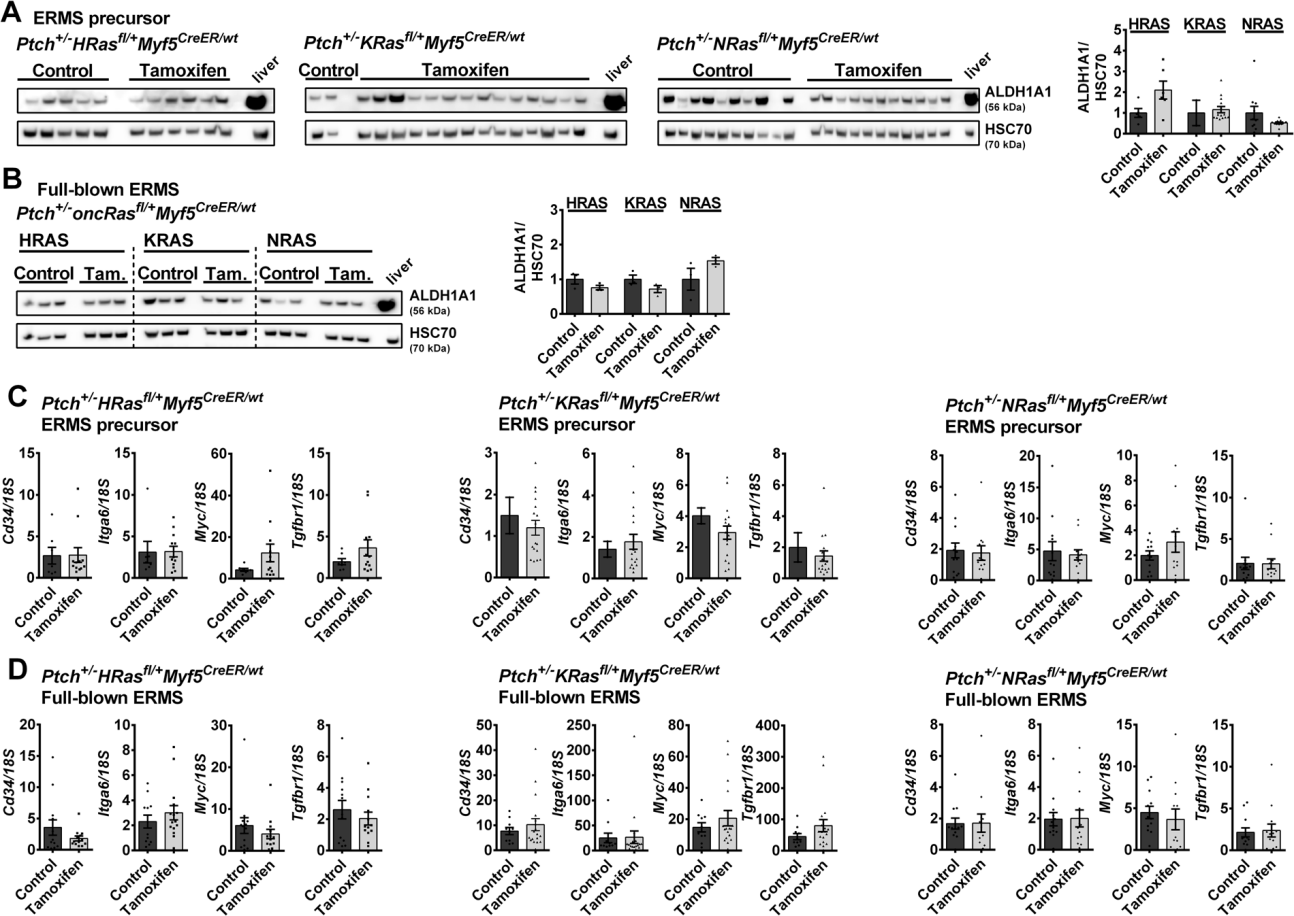
Expression of stem cell markers in oncRAS-expressing ERMS from *Ptch*^*+/****−***^ mice. **A**, **B** Representative western blots (left) and respective densitometric analyses (right) for ALDH1A1 protein levels of tamoxifen-treated *Ptch*^*+/****−***^*HRas*^*fl/+*^*Myf5*^*CreER/wt*^*, Ptch*^*+/****−***^*KRas*^*fl/+*^*Myf5*^*CreER/wt*^, or *Ptch*^*+/****−***^*NRas*^*fl/+*^*Myf5*^*CreER/wt*^ mice with oncRAS mutations induced at the precursor (**A**) or the full-blown stage (**B**) in comparison to control mice. **C**, **D** qRT-PCR analyses of *Cd34*, *Itga6*, *Myc*, and *Tgfbr1* in ERMS with oncRAS induction at the ERMS precursor stage (**C**) or at the full-blown ERMS stage (**D**) shown as fold expression of the same gene in normal muscle of the same mouse, which was set to 1. Dots: values from individual tumors. Statistical evaluation was done by non-parametric *t*-tests (Mann–Whitney). Bars: mean ± SEM.

To test if oncRAS also regulates the expression of CSC genes in ERMS of *Ptch*^*+/−*^ mice, the intratumoral protein level of ALDH1A1 was analyzed. ALDH1A1 protein expression was heterogeneous and did neither correlate with oncRAS induction at the precursor stage, (Fig. 7A) nor with induction at full-blown tumor stage (Fig. 7B). Equally, the CSC markers *Cd34*, *Itga6*, *Myc*, and *Tgfbr1* that were upregulated by oncRAS in human ERMS cell lines, showed no significant differences compared to the controls (Fig. 7C, oncRAS-expressing ERMS derived from precursor lesions; Fig. 7D, ERMS that had received the oncRAS mutation at the full-blown stage; please note that *Lin28b*, *Cxcl8*, and *Wwc1* were not detected in ERMS or skeletal muscle). Thus, in contrast to human ERMS cell lines, oncRAS mutations rather do not influence the expression of stem cell markers in ERMS of *Ptch*^*+/−*^ mice, at least not in the bulk of the tumors.

## Discussion

Our data show that oncRAS mutations are advantageous for specific ERMS precursor lesions (murine ERMS model) and ERMS cell lines (human ERMS model) and alter the expression of CSC markers in a context- and isoform-dependent manner. In addition, oncRAS can decrease GLI1/*GLI1* expression in cell lines derived from sporadic ERMS. Because concomitantly cellular proliferation was increased, the data suggest that HH signaling is not the main driver of growth of sporadic ERMS, although ERMS cell lines are sensitive to the GLI1/2 inhibitor GANT61 [38]. However, oncRAS might override the need for other growth stimuli such as GLI1, because it is a very potent proliferative stimulus. This also has been shown in medulloblastoma, in which oncHRAS circumvents HH pathway dependency, drives tumor growth, and enhances metastatic behavior [39].

In ERMS cell lines, the MEK-ERK axis of oncRAS is of great importance for inhibition of *GLI1*/GLI1 expression. This is similar to a report showing that the MEK-ERK arm is required for an oncKRAS-mediated block of *GLI1* expression in fibroblasts and pancreatic carcinoma cell lines [40]. Interestingly, this block needs DYRK1B. Because DYRK1B (i) is important for rhabdomyosarcoma growth [41], (ii) can block HH signaling [40], and (iii) is a novel ERK2 substrate [42], it is possible that ERK represses the HH pathway via DYRK1B. However, this is pure speculation and remains to be analyzed in the future.

In contrast, *Gli1* expression was not suppressed by oncRAS mutations in the *Ptch*^*+/−*^ model, which to some extent supports the importance of the Hh pathway for ERMS.

In the *Ptch*^*+/−*^ ERMS model and similar to ERMS cell lines, oncHRAS and oncKRAS enforced tumor proliferation when induced at the ERMS precursor stage. However, oncKRAS was more aggressive and additionally decreased ERMS-free survival. Together with the fact that induction of oncNRAS at the early tumor stage did not alter ERMS growth behavior, our results show that the three oncRAS isoforms can have different functions in ERMS pathogenesis.

When induced at the precursor stage in *Ptch*^*+/−*^ mice, oncNRAS did not influence ERMS growth and induced differentiation and myogenin expression. This is surprising, because in human ERMS oncNRAS mutations are rather associated with an aggressive phenotype and are the most frequent oncRAS mutations [10, 11]. In addition, the endogenous *NRAS*^*Q61H*^ mutation in human RD cells inhibits myogenic differentiation by repression of myogenin [43]. This discrepancy may reflect species-specific differences in tumor pathobiology. It is also possible that the *Nras*^*G12D*^ mutation is not functional in *Ptch*^*+/−*^ mice. However, this assumption is unlikely because oncNRAS-associated murine ERMS show elevated RAS activity (see Fig. S4A) and because the *Nras*^*G12D*^ allele induces malignancies in other models (e.g., see ref. [44]). Our data rather argue for the conclusion that oncRAS-associated processes differ from each other in dependency on their occurrence during tumor development. This hypothesis is supported by the fact that none of the oncRAS mutations influenced tumor growth when induced at the advanced tumor stage in the *Ptch*^*+/−*^ model. Therefore, it is possible that induction of the oncNRAS mutation at a different stage (e.g., at an earlier time point), when the prospective tumor cells are molecularly different and permissive to the respective mutation, may result in a more aggressive ERMS growth. This scenario would be similar to many other cancer-related mutations that can show cell type, cell differentiation and tumorigenesis-stage specificity (for review see ref. [45]).

Similar to LOH of 11p15.5, oncRAS mutations are generally considered as ERMS founding lesions [7]. However, our data on human cell lines show that oncRAS mutations also function as “advantageous mutations” for already established ERMS cells. In addition, the mutations seem to enlarge the ALDH^high^ populations that potentially belong to cancer-initiating cells in sarcoma [34, 46]. Furthermore, oncRAS mutations induce the expression of several CSC markers in RUCH-2 cells. Therefore, it is possible that oncRAS can enhance ERMS development and proliferation by pushing the cells into a CSC phenotype. However, this is hypothetical and needs verification.

We currently do not know why oncRAS mutations do not affect growth of full-blown ERMS in *Ptch*^*+/−*^ mice. Similar to human ERMS cell lines that are also derived from full-blown ERMS, ERMS of *Ptch*^*+/−*^ mice contain ALDH1A1^+^ subpopulations and express CSC markers, which however are not modulated by oncRAS. Again, this discrepancy may reflect species-specific differences in tumor pathobiology or could be related to active Hh signaling. Yet it is also well possible that the full-blown murine tumors grow independently of RAS signaling. We currently also do not know if ERMS precursor lesions of *Ptch*^*+/−*^ mice contain cells that could be specifically targeted by oncRAS. However, this seems likely because oncHRAS and oncKRAS germline mutations per se do not lead to ERMS, at least not in the mouse [14–17]. Therefore, both mutations must have affected growth of already existing ERMS precursor lesions in *Ptch*^*+/−*^ mice. This argues for the intriguing possibility that oncRAS mutations are not the ERMS-initiating event but are advantageous for already initiated ERMS lesions.

If oncRAS mutations are not the ERMS-initiating event, the alternative could be LOH of 11p15.5, which is much more common and occurs in almost all ERMS (e.g., 24/25 fusion-negative RMS described by ref. [7]). LOH of 11p15.5 is usually accompanied by uniparental di- to pentasomy [7] with loss of maternal genetic information and duplication of the paternal one, which results in IGF2 overexpression [47, 48]. Interestingly, LOH or uniparental disomy of 11p15.5 are also seen in ERMS from patients with Costello Syndrome or Noonan Syndrome [13, 49, 50]. Together with the facts that (i) onc*Ras* mutations in mice do not result in ERMS, (ii) almost all ERMS overexpress IGF2, and (iii) *Igf2* is indispensable for ERMS formation, at least in mice [51], it is possible that LOH of 11p15.5, and not an oncRAS mutation, is the ERMS-initiating event. Whether this is true or not remains to be analyzed in future studies.

## Materials and methods

### Cell lines

The human ERMS cell lines RUCH-2 and TE617.T were transduced with *pMSCVpuro* vector (Clontech, #634401) containing *RAS* sequences derived from *pCaggs-NRAS*^*G12V*^ [52], a *KRAS*^*G12V*^ plasmid [40] or *pBabe puro HRAS*^*G12V*^ (Addgene plasmid #905).

Source of cell lines, culture conditions, and detailed experimental procedures for standard methods (e.g., BrdU incorporation assay, WST-1 and Aldefluor assays, flow cytometry, xenografting, and analysis of gene or protein expression) are described in the Supplementary Material and Methods section.

### Animal experiments

Studies have been approved by the Lower Saxony State Office for Consumer Protection and Food Safety (file numbers 33.14.42502-04-13/1284, 33.9-42502-04-12/0805, and 33.14.42502-04-17/2534). Numbers of used animals are included in the respective figures or tables.

We used nude (Crl:NU(NCr)-*Foxn1*^*nu*^, Charles River), *Ptch*^*+/−*^ [35], *Myf5*^*CreER*^ [37], *Rosa26R-LacZ* (*R26R*, JAX stock #002073, [53]) mice, and HRAS (*FR-HRASG12V;* [16]), KRAS (*LSL-K-RASG12D*; [54]) or NRAS (*NRAS LSL-G12D* [44])—collectively named *oncRas*^*fl/+*^—mice for the studies. Detailed breedings, cre-recombination upon tamoxifen injection, tumor monitoring, µCT measurements, and immunohistochemical analyses are described in the Supplementary Material and Methods section. Utilized oligonucleotides and antibodies are depicted in Supplementary Tables S1 and S2, respectively.

### Statistical analyses

Statistical tests done by Microsoft^®^ Excel^®^ 2016 or GraphPad Prism 6 are given in the respective figure legends. Data were considered significant when *p* < 0.05. All tests were two-sided and *p*-values were not corrected for multiple testing.

## Supplementary information

Supplemental Material
