# Supplementary material for: Context-dependent modulation of aggressiveness of pediatric tumors by individual oncogenic RAS isoforms
Source: Oncogene. 2021 Jun 25;40(31):4955–66. doi: 10.1038/s41388-021-01904-4 (PMC8342309; doi:10.1038/s41388-021-01904-4)
Supplement: Supplementary file 1 — Supplemental Material [file 41388_2021_1904_MOESM1_ESM.docx]

**Supplementary Material**

**Supplementary Methods**

**Cell culture**

The human ERMS cell lines RUCH-2 (gift from Beat Schäfer, University Zürich, Switzerland) and RD (from ATCC: #CCL-136) were cultured in DMEM. TE617.T cells (from ATCC: #CRL-7774) were cultured in DMEM without pyruvate. All media were supplemented with 10% FCS and 1% penicillin/streptomycin (Thermo Fisher Scientific, Waltham, MA, USA). RUCH-2 and TE617.T are wildtype for *RAS* [[1](#_ENREF_1), [2](#_ENREF_2)], whereas RD cells have a *NRAS^Q61H^* mutation [[3](#_ENREF_3)]. Testing for *Mycoplasma* of stable transduced cell lines by RT-PCR was done regularly and cells were kept in culture for less than 20 passages.

*RAS* sequences from *pCaggs-NRAS^G12V^* [[4](#_ENREF_4)], a *KRAS^G12V^* plasmid [[5](#_ENREF_5)] or *pBabe puro HRAS^G12V^* (a gift from William Hahn; Addgene plasmid #905) were cloned into the *pMSCVpuro* vector (Clontech, Mountain View, CA, USA; #634401). The resulting expression vectors *pMSCVpuro*, *pMSCVpuro-HRAS^G12V^*, *pMSCVpuro-KRAS^G12V^* or *pMSCVpuro-NRAS^G12V^* were utilized to generate oncRAS-expressing cells by retroviral transduction. Sequences and correct fragment insertion was verified by Sanger sequencing and RT-PCR-based cDNA amplification. Primers are listed in Supplemental Table S1.

DMSO (< 1 %, Sigma-Aldrich, St. Louis, MO, USA) was used as solvent for PI-103 (3 µM end concentration, 3 mM stock concentration; Axxora, Lörrach, Germany), U0126 (10 µM end concentration, 10 mM stock concentration, Cell Signaling, Danvers, MA, USA) and SCH772984 (0.5 µM end concentration, 10 mM stock concentration, Selleck Chemicals, Houston, TX, USA). The respective drugs were applied for 24 h to cells that grew at normal log phase conditions in 10% FCS (please note that starvation had no impact on the results). Gene and protein expression analyses were performed as described below.

Knockdown of ERK1 and/or ERK2 was achieved by transient transfection of small interfering RNA (siRNA) pools, which were FlexiTube GeneSolution GS5595 for MAPK3/ERK1 and FlexiTube GeneSolution GS5594 for MAPK1/ERK2 from Qiagen (Hilden, Germany). 100 nM of each siRNA pool was used alone or in combination. Scrambled siRNA (AllStars negative, Qiagen) was used as negative control siRNA. 1,5 x 10^5^ RMS cells of each well of a 6-well plate were transfected with 100 nM siRNA pool using the NeonTransfection System (Invitrogen, Carlsbad, CA, USA) according to the manufacturer’s instructions and the following transfection conditions: 2 pulses at 1,000 V and a pulse width of 30 ms for RD cells and 1 pulse at 1,200 V and a pulse width of 40 ms for RUCH-2 cells. 48 h after transfection the cells were additionally incubated for 24 hours with PI-103.

Cell proliferation was measured by a BrdU-incorporation ELISA (Roche Diagnostics, Basel, Switzerland) according to the manufacturer’s instructions. BrdU-incorporation was analyzed after cellular attachment (24 h) and incubation with 10 µM BrdU (additional 24 h – 72 h). WST-1 assays were performed in parallel to BrdU-incorporation assays with identical incubation times. For analysis of cellular viability by WST-1, cells were stained at 37 °C for 3 h using a 1:25 dilution of WST-1 reagent (Sigma-Aldrich). Signal intensity was measured in a microplate reader (BioTek Instruments, Winooski, VT, USA) at wavelengths of 450 nm versus 655 nm. Results are presented relative to the control cells (100%).

**Flow cytometry**

The Aldefluor assay (Stemcell Technologies, Vancouver, Canada) was performed according to the manufacturer’s instructions. Precisely, 1 x 10^6^ cells were stained with the substrate for 40 min in an incubator and addition of the specific ALDH inhibitor (DEAB) served as negative control. Dead cells were excluded by 7-AAD staining (Becton Dickinson, Holdrege, NE, USA; 0.125 µg/0.5 x 10^6^ cells). Data from 2.5 x 10^5^ stained cells were recorded on a FACS Canto II with the FacsDiva™ software (both Becton Dickinson) and analyzed using FlowJo software (Treestar Inc, Ashland, OR, USA). The percentage of ALDH^high^ cells was calculated by subtraction of living ALDH^high^ cells in the DEAB control sample from living ALDH^high^ cells in the test sample.

**Xenograft models**

The experiments have been approved by the Lower Saxony State Office for Consumer Protection and Food Safety (file number 33.14.42502-04-13/1284).

Aliquots of 2 x 10^6^ (TE617.T) or 9 x 10^6^ (RUCH-2) viable pMSCV- or oncRAS-transduced cells in 200 µl PBS (TE617.T) or Matrigel (Corning, New York, USA)/PBS suspension (1:1; RUCH-2) were injected subcutaneously into the left and right flank of 8- to 13-week old nude mice (Crl:NU(NCr)-Foxn1^nu^, Charles River), respectively. Tumor size was measured every second day by caliper and tumor volumes were calculated by: tumor volume = ½ x tumor length x tumor with x tumor hight [[6](#_ENREF_6)]. At the end of the study, tumors were dissected, weighed and samples were frozen or fixed in a 4% paraformaldehyde for paraffin embedding.

Please note that 5×10^6^ transplanted RUCH-2 cells do not form tumors until 3.5 months. These tumors are 4–6 mm in diameter and about a quarter of the size of tumors induced by RD cells [[7](#_ENREF_7)]. Therefore, we decided to transplant 9 x 10^6^ cells of this cell line.

**Genetically engineered mouse models**

The experiments have been approved by the Lower Saxony State Office for Consumer Protection and Food Safety (file numbers 33.9-42502-04-12/0805 and 33.14.42502-04-17/2534).

*Ptch^+/-^* mice harbor a heterozygous *Ptch* germline mutation (for generation see [[8](#_ENREF_8)]). *Myf5^CreER^* mice were produced by a knock-in strategy [[9](#_ENREF_9)]. Both strains were backcrossed onto a Balb/cJ background to achieve a high RMS susceptibility [[10](#_ENREF_10), [11](#_ENREF_11)].

*Rosa26R-LacZ* mice (*R26R,* JAX stock #002073, [[12](#_ENREF_12)]), which carry a Cre-inducible *LacZ* gene under the endogenous *Rosa26* promoter, and mice conditionally expressing oncogenic HRAS (*FR-HRASG12V;* [[13](#_ENREF_13)]), KRAS (*LSL-K-RASG12D*; [[14](#_ENREF_14)]) or NRAS (*NRAS LSL-G12D* [[15](#_ENREF_15)]) were on a pure C57BL/6 background. Cre recombination by tamoxifen injection (see below) induced removal of a floxed stop cassette in *NRas^fl/+^* and *KRas^fl/+^* mice or of the floxed wildtype *Hras* locus in *HRas^fl/+^* mice induced the expression of oncogenic *Ras* variants.

For lineage tracing *Ptch^del/+^R26R^tg/-^* *Myf5^CreER/wt^* mice were generated and Cre-mediated recombination was induced by tamoxifen injection (see below). For this purpose, *Ptch^+/-^* mice were bred to *R26R* mice and the resulting *Ptch^+/-^R26R^tg/-^* mice were crossed to *Myf5^CreER/CreER^* mice.

For tumor studies *Ptch^+/-^* mice were bred to mice expressing oncogenic *Ras* variants (collectively named *oncRas^fl/+^*). The resulting *Ptch^+/-^oncRas^fl/+^* mice were bred to *Myf5^CreER/wt^* or *Myf5^CreER/CreER^* mice to induce oncRAS mutations in ERMS. *Ptch^+/-^oncRas^fl/+^Myf5^CreER/wt^* offspring were injected intraperitoneally with 1 mg tamoxifen (10 mg/ml in sterile ethanol:sun flower seed oil, 1:25, Sigma-Aldrich) on 5 consecutive days [[16](#_ENREF_16)] at an age of 4 weeks or when tumors had occurred. Uninjected (age of 4 weeks) or solvent injected (tumor-bearing) *Ptch^+/-^oncRas^fl/+^Myf5^CreER/wt^* littermates served as controls. Destribution in groups was performed randomely. Tamoxifen-treated *Ptch^+/-^* mice littermates were used to investigate drug-intrinsic effects. Cohort sizes were estimated with G*Power. Both female and male mice were used in the study.

Mice were monitored weekly for palpable tumors for at least 200 days or until an termination condition was reached. All sacrificed mice were examined for non-palpable tumors (genotypes of the mice were blinded). The identity of the tumors was confirmed by hematoxylin and eosin (H&E) staining. Recombination at the targeted *Ras* loci was estimated by PCR on genomic DNA (*Kras, Nras*) or cDNA (*Hras*) derived from skeletal muscle (SM) and ERMS. DNA extraction was performed with STE buffer (100 mM NaCl, 50 mM Tris, 1 mM EDTA, 1% SDS, 250 µg proteinase K, pH8, 55°C) followed by precipitation in ethanol or with the DNA FFPE Tissue Kit (Qiagen, Hilden, Germany) according to manufacturer’s protocol.

All primers used for genotyping, sequencing of genomic DNA, recombination assays and detection of basal *Ras* expression are listed in Table S1.

**µCT tumor measurement**

ERMS growth in *Ptch^+/-^oncRas^fl/+^Myf5^CreER/wt^* mice was assessed by *in vivo* µCT using a low-dose laboratory animal CT system (QuantumFX, Perkin Elmer Health Sciences, Boston, MA, USA) [[17](#_ENREF_17)] directly before tamoxifen treatment and 7 weeks thereafter. For visualization, Imeron 300 (Bracco Imaging, Milan, Italy) [[18](#_ENREF_18)] was injected into the tail vein (5 µl/g body weight) or retrobulbar (50 µl/mouse) of anaesthetized mice approximately 30 sec prior imaging. Data sets were reconstructed with a voxel size of 80 μm, the tumor size was analyzed using the QuantumFX simple viewer software and tumor volume was calculated by assuming an ellipsoid shape as described for xenograft models.

**Quantitative real time PCR (qRT-PCR) and Sanger-sequencing**

Total mRNA from cell lines or mouse tissue was extracted with TRIzol (Thermo Fisher Scientific) and 2 µg mRNA were reverse transcribed [[11](#_ENREF_11)].

Gene expression was analyzed by RT-PCR or by SYBR®-Green (Invitrogen, Carlsbad, CA, USA or Qiagen) based qRT-PCR on the 7900HT sequence detection system (Applied Biosystems, Foster City, CA, USA). qRT-PCR data were analyzed by the standard curve method for absolute quantification and are shown relativ to the amplification of *18S* rRNA. All utilized primers are described in Table S1.

Gene expression analyses from cell culture experiments summarize at least three independent experiments, each performed and measured in triplicates. All available samples from murine tissue were analyzed and measured in technical triplicates.

Sequencing was done on an ABI 3500 XL sequencer (Applied Biosystems) using the primers given in the Table S1.

For RT² Profiler PCR Arrays (PAHS-176Z, Qiagen) 600 µg of RNA were reversed transcribed with the RT^2^ First Strand Kit (Qiagen) according to the manufacturer’s protocol. Measurements were performed with the QuantStudio5 Real-Time PCR system (Applied Biosystems). Data were analyzed using the Qiagen web portal GeneGlobe. Heatmaps were generated with Morpheus (https://software.broadinstitute.org/morpheus).

**Immunohistochemistry and X-Gal staining**

For antibody staining, paraffin-embedded tumor samples were sectioned at 5 µm and stained with primary and secondary antibodies as listed in Table S2. The number of Ki67^+^ and SOX2^+^ nuclei was determined on 6 to 10 and 3 randomly chosen tumor areas, respectively. For counting, stained tissue sections were documented at 100- (Ki67) or 200-fold magnification on a microscope (Olympus BX 60) with software cellSens Dimension (Olympus, Shinjuku, Japan).

β-galactosidase activity was analyzed by X-Gal staining of cryo-embedded tissue sections (compare [[19](#_ENREF_19)]) and the stainings were documented at 200-fold magnification on a microscope (Olympus BX 60) equipped with software cellSens Dimension (Olympus).

**Protein isolation and Western blot**

Cells or homogenized murine tisse samples were lysed in lysis buffer or modified RIPA buffer, respectively [[20](#_ENREF_20)]. For subcellular fractionation cells were lysed in fractionation buffer with 2 mM Dithiothreitol and a protease inhibitor cocktail (Roche Diagnostics). After centrifugation, the cytosolic fraction was collected from the supernatant and the pellet was lysed in nuclear lysis buffer with protease inhibitor cocktail to obtain the nuclear fraction (modified according to <http://www.abcam.com/protocols/subcellular-fractionation-protocol>).

Active RAS was detected using the Ras pull-down activation assay kit according to the manufacturer’s protocol (Cytoskeleton Inc., Denver, CO, USA). In short, and as described on the manufacturers web page, the assay uses the Ras-binding domain (RBD) of the Ras effector kinase Raf1, which specifically binds to the GTP-bound, active form of Ras proteins with high affinity. In the assay, the Raf-RBD is in the form of a GST fusion protein, which allows for a pull-down the Raf-RBD/GTP-Ras complex with glutathione affinity beads. The amount of activated Ras was determined by a Western blot using the Ras specific mAb mouse anti-pan-Ras antibody (see Table S2), which was provided with the kit.

Proteins were separated on SDS gels (Invitrogen) and blotted onto nitrocellulose membranes (GE Healthcare Life Sciences, Chicago, IL USA). After blocking in 5% dry milk in TBST (150 mM NaCl, 10 mM Tris/HCl, 0.5% Tween-20), membranes were incubated with primary antibodies (compare Table S2) overnight at 4 °C. Signals were visualized by ECL (GE Healthcare) after incubation with HRP-conjugated secondary antibodies on a FluorChem Q (Protein Simple, San Jose, CA, USA) or Azure c300 (Azure Biosystems, Dublin, CA, USA) imaging system. Pictures were processed with Adobe Photoshop and analyzed with ImageJ.

**Supplementary Tables**

**Table S1: Utilized oligonucleotides**

| **Oligonucleotides used for genotyping, recombination assays and sequencing** | | | | |
| --- | --- | --- | --- | --- |
| **Primer name** | **Primer sequence (5’ -> 3’ orientation)** | | **Transcript** | **Reference** |
| **Primer for genotyping** | | | | |
| Exon 7-F | AGGAAGTATATGCATTGGCAGGAG | | 950 bp (mutated *Ptch*) | [[21](#_ENREF_21)] |
| Neo-R | GCATCAGAGCAGCCGATTGTCTG | |  |  |
| mPTCNx_f | TGGTAATTCTGGGCTCCCGT | | 445 bp (wt *Ptch*) | [[21](#_ENREF_21)] |
| mPTCwt_r.2 | ACACAACAGGGTGGAGACCACT | |  |  |
| eCreRasF | GCCATCCCTCGCGTTCCTGTAGTC | | 622 bp (wt *Hras*), 667 bp (mutated *Hras*) | [[13](#_ENREF_13)] |
| eCreRasR | CCTGCCCCACCTGCCAATGAGAAG | |  |  |
| Kras-WT-UP1 | CACCAGCTTCGGCTTCCTATT | | 270 bp (wt *Kras*), 170 bp  (mutated *Kras*) | [[14](#_ENREF_14)] |
| Kras-URP_Lp1 | AGCTAATGGCTCTCAAAGGAATGTA | |  |  |
| KrasG12Dmut_UP | CCATGGCTTGAGTAAGTCTGC | |  |  |
| mNRas-WT-For | AGACGCGGAGACTTGGCGAGC | | 487 bp (wt *Nras*), 345 bp (mutated *Nras*) | [[15](#_ENREF_15)] |
| mNRas-WT-Rev | GCTGGATCGTCAAGGCGCTTTTCC | |  |  |
| mNRas-Mut-Rev | AGCTAGCCACCATGGCTTGAGTAAGTCTGCA | |  |  |
| cF | GCATTTCTGGGGATTGCTTA | | 241 bp (*Myf5CreER)* | [[9](#_ENREF_9)] |
| cR | CCCGGCAAAACAGGTAGTTA | |  |  |
| CK382 | ACCCTCCAGCTCCAGACTTATC | | 454 bp (wt) Myogenic factor 5 *(Myf5)* | [[9](#_ENREF_9)] |
| CK383 | CCCTGTAATGGATTCCAAGCTG | |  |  |
| Rosa1 | AAAGTCGCTCTGAGTTGTTAT | | 500 bp (wt), 250 bp (mutated) | [[12](#_ENREF_12)] |
| Rosa2 | GCGAAGAGTTTGTCCTCAACC | |  |  |
| Rosa3 | GGAGCGGGAGAAATGGATATG | |  |  |
| **Primer for recombination assays** | | | | |
| mHRasG12VrelF1 | TGGGGCAGGAGCTCCTGGATT | | 302 bp (*Hras* fragment) |  |
| mHRasG12VrelR1 | GGTGTTGTTGATGGCAAATAC | |  |  |
| Kras-WT_UP1 | CACCAGCTTCGGCTTCCTATT | | 270 bp (wt *Kras*), 304 bp (recombined *Kras*) | [[14](#_ENREF_14)] |
| Kras-URP_Lp1 | AGCTAATGGCTCTCAAAGGAATGTA | |  |  |
| mNRas-WT-For | AGACGCGGAGACTTGGCGAGC | | 487 bp (wt *Nras*), 521 bp (recombined *Nras*) | [[15](#_ENREF_15)] |
| mNRas-WT-Rev | GCTGGATCGTCAAGGCGCTTTTCC | |  |  |
| **Primer for sequencing of murine tumor tissue** | | | | |
| Kras_seq fwd2 | AGGCCTGCTGAAAATGACTG | | *Kras*, Cds, position 30-290 |  |
| Kras_seq rev3 | AGGAGTCCTCTATCGTAGGGT | | *Kras*, Cds, position 1-70 |  |
| Nras Exon1_seq rev1 | AAGTGAGGATAAGGGCCAGG | | *Nras,* exon 1, position 1–125 |  |
| Nras Exon1_seq fwd2 | CAAACTGGTGGTGGTTGGAG | | *Nras,* exon 1, position 100-128 |  |
| Nras Exon2_seq fwd1 | CCACCACCTCCTCACTCTTT | | *Nras,* exon 2, position 10-179 |  |
| Nras Exon2_seq rev2 | CCTTCGCCTGTCCTCATGTA | | *Nras,* exon 2, position 1-60 |  |
| Nras Exon3_seq fwd1 | ACTCCTCCAGAAAGCTTGCT | | *Nras,* exon 3, position 1-160 |  |
| Nras Exon3_seq rev1 | ACTCACACTTGCTTGAATCTCT | | *Nras,* exon 3, position 1-155 |  |
| Nras Exon4_seq rev1 | GCTGGCACTATTCTGAACTGC | | *Nras,* exon 3, position 1-124 |  |
| Hras Exon1_seq fwd1 | AAGTGTGCTTCTCATTGGCA | | *Hras,* exon 1, position 15-152 |  |
| Hras Exon2_seq fwd1 | AAGCCGTGTTGTTTTGCAG | | *Hras,* exon 2, po-sition 25-87, 93-179 |  |
| Hras Exon2_seq rev1 | GAACCTTCCTCACGTGTGC | | *Hras,* exon 2, position 1-179 |  |
| Hras Exon3_seq fwd1 | CTCATATCCACCCCATCCCC | | *Hras,* exon 3, position 25-160 |  |
| Hras Exon3_seq rev1 | GGGTGCGGTAAATCCTTGAC | | *Hras,* exon 3, position 1-160 |  |
| Hras Exon4_seq rev1 | GGGAGCAAGGACATCAGAGT | | *Hras,* exon 4, position 1-126 |  |
| **Primer for sequencing and plasmid identification in cell culture experiments** | | | | |
| pMSCVpuroseq-F | CCCTTGAACCTCCTCGTTCGACC | | 349 bp (empty vector) | Addgene |
| pMSCVpuroseq-R | GAGACGTGCTACTTCCATTTGTC | |  | Addgene |
| pHRasID-Rev | TCATCCGAGTCCTTCACCCGTT | | +pMSCVpuroseqF: 453 bp (oncHRAS fragment) |  |
| pKRasID-Rev | CCTCATGTACTGGTCCCTCATT | | +pMSCVpuroseqF: 596 bp (oncKRAS fragment) |  |
| pNRasID-Rev | GCCTTCGCCTGTCCTCATGTATTG | | +pMSCVpuroseqF: 319 bp (oncNRAS fragment) |  |
| **Primer used for semi-quantitative PCR** | | | | |
| **Primer name** | **Primer sequence** | **Primer location** | **Transcript** | **Amplicon size** |
| mHras_tqF2 | CAGCCAAGACCCGGCAG | Exon 4 | *Hras* | 150 bp |
| mHras_tqR2 | CCTGAGCCTGGTGTCAGGA | Exon 5 |  |  |
| mKras_tqF2 | AGCGCCTTGACGATACAGC | Exon 2 | *Kras* | 113 bp |
| mKras_tqR2 | TCCAAGAGACAGGTTTCTCCATC | Exon 3 |  |  |
| mNras_tqF1 | CACAAAGCAAGCCCACGAAC | Exon 4 | *Nras* | 124 bp |
| mNras_tqR1 | TCGGTACTGGCGTATCTCCC | Exon 5 |  |  |
| **Primer used for qRT-PCR** | | | | |
| **Primer name** | **Primer sequence** | **Primer location** | **Transcript** | **SYBR Green** |
| **Primer for human and murine sequences** | | | | |
| 18S-fwd | CGCAAATTACCCACTCCCG | Exon 1 | *18S* rRNA | Qiagen, Invitrogen |
| 18S-rev2 | TTCCAATTACAGGGCCTCGAA | Exon 1 |  |  |
| **Primer for human sequences** | | | | |
| HsaGli1tqF | AGCTACATCAACTCCGGCCA | Exon 11 | *GLI1* | Invitrogen |
| HsaGli1tqR | GCTGCGGCGTTCAAGAGA | Exon 12 |  |  |
| hHPRT1-F1 | TGGCGTCGTGATTAGTGATG | Exon 1 | *HPRT* | Invitrogen |
| hHPRT1-R1 | CGAGCAAGACGTTCAGTCCT | Exon 3 |  |  |
| hsC-MYCF | GTGCTCCATGAGGAGACA | Exon 3 | *MYC* | Invitrogen |
| hsC-MYCR | AGCCTGCCTCTTTTCCA | Exon 4 |  |  |
| **Primer for murine sequences** | | | | |
| mGli1-tqF | TACATGCTGGTGGTGCACATG | Exon 9 | *Gli1* | Qiagen |
| mGli1-tqR | ACCGAAGGTGCGTCTTGAGG | Exon 10 |  |  |
| Gli2-RT-PCR-F | GGTCATCTACGAGACCAACTGC | Exon 8 | *Gli2* | Qiagen |
| Gli2-RT-PCR-R | GTGTCTTCAGGTTCTCCAGGC | Exon 9 |  |  |
| mPtc10 | TACAGTCCGGGACAGCATACC | Exon 5 | *Ptch1* | Qiagen |
| mPtc11R | GTACCCATGGCCAACTTCGGCTTT | Exon 6 |  |  |
| mHhipF.1 | GGAGCCTTACTTGGACATTCACAA | Exon 4 | *Hhip* | Qiagen |
| mHhipR.2 | ACCGTTCCTGGTTGGTGGTATAA | Exon 5 |  |  |
| MyoD-ScerjF | GCAATGCACTGGAGTTCG | Exon 2 | *MyoD* | Qiagen |
| MyoD-ScerjR | ACGATGGACGTAAGGGAGTG | Exon 3 |  |  |
| MyogeninScerjF | GCAATGCACTGGAGTTCG | Exon 2 | *Myogenin* | Invitrogen |
| MyogeninScerjR | ACGATGGACGTAAGGGAGTG | Exon 3 |  |  |
| MHC tq F | AACACGAAGCGTGTCATCCAGTA | Exon 7 | *Myosin heavy chain* | Qiagen |
| MHC tq R | GTCTCGATGTCAGCAGATGCCAG | Exon 8 |  |  |
| mTropotF | GAGGATGAACTAGCAACCATGCA | Exon 2 | *Tropomyosin 3* | Qiagen |
| mTropotqR | CCAGCTCCTCTTCAACCAGCT | Exon 3 |  |  |
| mItga6_tq_F4 | CAGCGAAGGCAAAAGTGGTT | Exon 18 | *Itga6* | Invitrogen |
| mItga6_tq_R4 | GTTGCTGTGCCGAGGTTTTT | Exon 19 |  |  |
| mMyc_tq_F1 | CCTTTGGGCGTTGGAAACC | Exon 1 | *Myc* | Qiagen |
| mMyc_tq_R1 | CGTCGCAGATGAAATAGGGCT | Exon 2 |  |  |
| mCD34_tq_F2 | TGAGATGACATCACCCACCG | Exon 3 | *Cd34* | Invitrogen |
| mCD34_tq_R2 | GCCAACCTCACTTCTCGGAT | Exon 4 |  |  |
| mTgfbr1_tq_F3 | AGAGCGTTCATGGTTCCGAG | Exon 4 | *Tgfbr1* | Invitrogen |
| mTgfbr1_tq_R3 | GCTGCGTCCATGTCCCATT | Exon 5 |  |  |

Abbreviations: bp, base pairs; wt, wildtype

**Table S2:** **Primary and secondary antibodies for, immunohistochemistry, flow cytometry and Western Blot**

| **Primary antibodies for IHC** | **dilution** | **antigen retrieval** |
| --- | --- | --- |
| mAb rabbit anti-Ki67 (Clone B56), Becton Dickinson (#556003; RRID:AB_396287) | 1:50 | Citrate pH 6.0; microwave |
| mAb rabbit anti-Myosin Heavy Chain (Clone JF097-7), Invitrogen (#MA5-32555; RRID:AB_2809832) | 1:100 | Citrate pH 6.0; microwave |
| pAb rabbit anti-Tropomyosin 3, Abcam (ab180813) | 1:100 | Citrate pH 6.0; microwave |
| mAb rat anti SOX2, eBioscience (14-9811) | 1:100 | Citrate pH 6.0; microwave |
| **Secondary antibodies for IHC** | **dilution** | |
| EnVision ™/HRP*, rabbit/mouse, Dako (#K5007) | undiluted | |
| **Primary antibodies for Western blot** | **dilution** | |
| mAb mouse anti-α-Tubulin (Clone DM1A), Dianova (#DLN-009993) | 1:10000 | |
| mAb mouse anti-AKT (55/PKBa/Akt), Becton Dickinson (#610861; RRID:AB_398180) | 1:1000 | |
| mAb rabbit anti-p-AKT (Ser473), Cell Signaling Technology (#193H12; RRID:AB331168 ) | 1:1000 | |
| mAb mouse anti-p-ERK (Thr202/Tyr204), Cell Signaling Technology, (#9106; RRID:AB_331768) | 1:1000 | |
| pAb rabbit anti-ERK, Sigma Aldrich (#M5670; RRID:AB_477216) | 1:1000 | |
| pAb rabbit anti-GLI1 (V812), Cell Signaling Technology, (#2534; RRID:AB_2294745) | 1:750 | |
| mAb mouse anti-HSC70 (B-6), Santa Cruz Biotechnology (#sc-7298; RRID:AB_627761) | 1:10000 | |
| pAB rabbit anti-Lamin B1, Cell Signaling Technologies (#9087; RRID:AB_10896336) | 1:1000 | |
| pAB rabbit anti-Ras, Cell Signaling Technologies (#3965; RRID:AB_2180216) | 1:1000 | |
| mAb mouse anti-pan-Ras, Cytoskeleton Inc. (to detect RAS-GTP and pan-RAS; provided by the Ras Pull-down Activation Assay #BK008;) | 1:250 | |
| pAb goat anti-ALDH1A1 (Ser2-Ser501), R&D Systems (#AF5869; RRID:AB_2044597) | 1:250 | |
| **Secondary antibodies for Western blot** | **dilution** | |
| pAb goat anti-rabbit IgG/HRP, Dianova Jackson Immunoresearch (#111-035-045) ** | 1:10000 | |
| pAb rabbit anti-mouse IgG/HRP, Dianova Jackson Immunoresearch (#315-035-003) ** | 1:10000 | |
| pAb donkey anti-goat IgG/HRP, Dianova Jackson Immunoresearch (#705-035-003) ** | 1:10000 | |

* antibody binding was visualized using DAB+ (EnVision+ system-HRP, Dako, Santa Clara, USA) or aminoethylcarbazol as chromogen.

** signals were visualized using the ECL plus detection system (GE Healthcare Life Sciences, Chicago, USA).

Abbreviations: HRP, horseradish peroxidase; IHC, immunohistochemistry; mAb, monoclonal antibody; pAb, polyclonal antibody; RRID, Research Resource Identifier

**Supplementary Figures**

**

**

**Figure S1: Characteristics of oncRAS-expressing ERMS cell lines.**

**A, B, C)** Representative Western blots (n=2) for pERK/ERK and pAKT/AKT levels in PI-103-, U0126- or SCH772984-treated HRAS, KRAS or NRAS-expressing RUCH-2 (A), RD (B) and TE617.T (C) cells compared to respective pMSCV control cells. The right panel in B) shows a qRT-PCR analysis of *GLI1* mRNA expression (n=3) of SCH772984-treated RD cells. **D)** Representative light microscopic images of stably transduced RUCH-2 (upper row) and TE617.T cell (lower row). Scale bar: 100 µm. In all Western blot analyses HSC70 served as reference protein. Bars show mean + SEM. * significant by Mann-Whitney test in comparison to solvent-treated cells. ** p<0.01. Each sample for qRT-PCR analysis was measured in technical triplicates and normalized to *18S rRNA* (18S). The data are shown as fold induction over the expression level of solvent-treated cells, which was set to 1. DMSO-treated cells (1 µl/ml) served as control; PI-103: 3 µM; U0126: 10 µM; SCH772984: 0.5 µM.


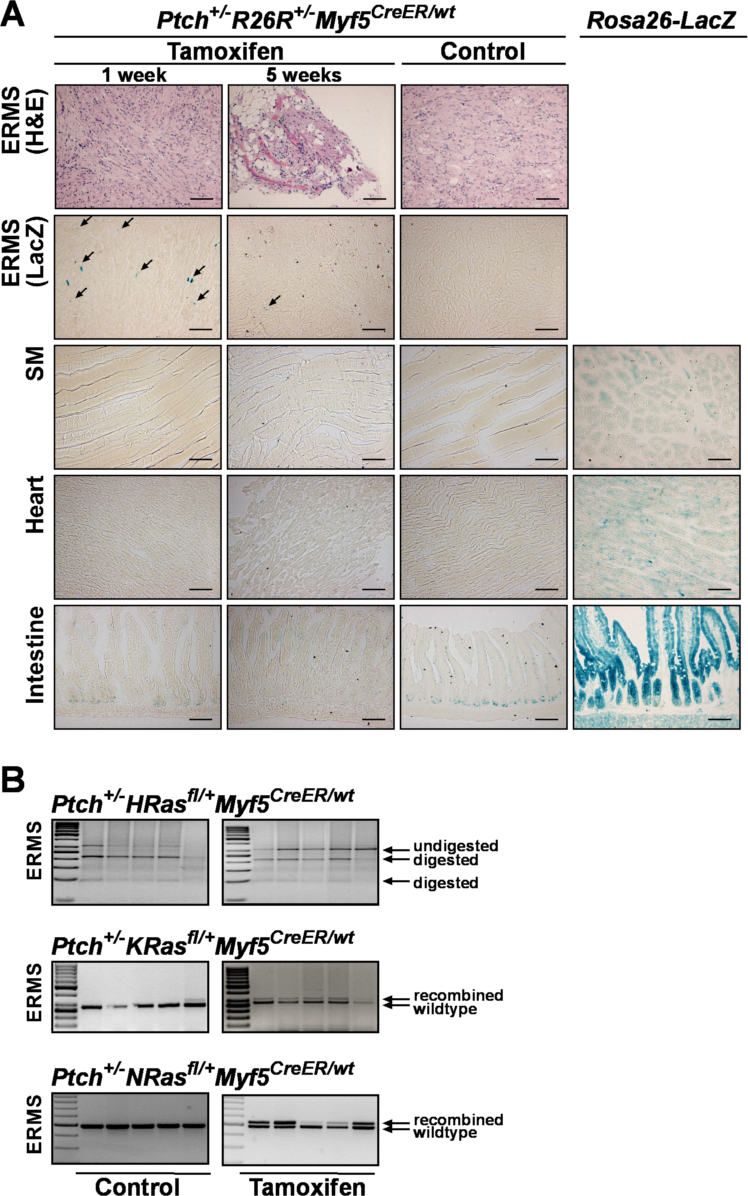


**Figure S2: Expression of *Ras* isoforms and *Myf5^CreER^* driver activity at the oncRAS loci in ERMS of *Ptch^+/-^* and *Ptch^+/-^R26R^+/-^Myf5^CreER/wt^* or *Ptch^+/-^oncRAS^fl/+^Myf5^CreER/wt^* mice, respectively.**

**A)** Representative LacZ stainings of different tissues from *Ptch^+/-^R26R^+/-^Myf5^CreER/wt^* (n=4 for tamoxifen-treated *Ptch^+/-^R26R^+/-^Myf5^CreER/wt^* mice, n = 2 for *Ptch^+/-^R26R^+/-^Myf5^CreER/wt^* control mice) and *Rosa26-LacZ* (positive control) mice*.* Cre activity was activated by intraperitoneally tamoxifen injection when the animals had developed a palpable tumor. Sections of ERMS, skeletal muscle (SM), heart and intestine were analyzed 1 or 5 weeks after tamoxifen injection by X-Gal staining. Besides moderate staining of the intestine, recombination has been cleary observed in ERMS. Although the staining was heterogeneous (compare [[9](#_ENREF_9)]), it confirmed that the *Myf5^CreER^*-driver is active in ERMS. ERMS were additionally stained with H&E. ERMS of solvent treated *Ptch^+/-^R26R^+/-^Myf5^CreER/wt^* mice were analyzed as controls. Scale bars represent 100 µm. Arrows indicate specific LacZ staining in the tumor. **B)** Representative RT-PCR analyses for cre-mediated recombination in ERMS of control or tamoxifen-treated *Ptch^+/-^HRas^fl/+^Myf5^CreER/wt^* (upper panel), *Ptch^+/-^KRas^fl/+^Myf5^CreER/wt^* (middle panel) or *Ptch^+/-^NRas^fl/+^Myf5^CreER/wt^* (lower panel) mice. The recombination assay for *Hras* was done with PCR-amplified cDNA by enzymatic digestion with BpmI [[13](#_ENREF_13)]. Successful recombination is indicated by a stronger undigested fragment in comparison to digested fragments. The recombination assays for *Kras* and *Nras* were done with gDNA and a double band indicates efficient recombination, whereas a single band indicates no recombination and shows the wildtype allele only.

**

**

**Figure S3: Tumor development in *Ptch^+/-^* mice upon tamoxifen application, Hh signaling activity and immunohistological analyses of tropomyosin and myosin heavy chain (MyHC) upon oncRAS induction at the ERMS precursor stage.**

**A)** ERMS development in *Ptch^+/-^* mice injected with tamoxifen at an age of 4 weeks. Shown is the overall survival (first panel), the ERMS-free survival (including only palpable ERMS, second panel) the total ERMS incidence (including both palpable and non-palpable ERMS, third panel) and the percentage of Ki67^+^ nuclei in ERMS tissue sections (forth panel). 24 control and 26 tamoxifen-treated *Ptch^del/+^* mice were used for ERMS monitoring. For Ki67^+^ staining, 19 ERMS from 10 control and 15 ERMS from 12 tamoxifen-treated *Ptch^+/-^* mice were analyzed. **B, C, D)** qRT-PCR analyses of the Hh signaling molecules *Gli1*, *Gli2*, *Ptch1* and *Hhip* in ERMS of (B) *Ptch^+/-^HRas^fl/+^Myf5^CreER/wt^*, (C) *Ptch^+/-^KRas^fl/+^Myf5^CreER/wt^* or (D) *Ptch^+/-^NRas^fl/+^Myf5^CreER/wt^* mice. For analysis, ERMS and skeletal muscle tissue samples were isolated from 7 control and 12 tamoxifen-treated *Ptch^+/-^HRas^fl/+^Myf5^CreER/wt^* mice, 2 control and 17 tamoxifen-treated *Ptch^+/-^KRas^fl/+^Myf5^CreER/wt^* and 12 control and 12 tamoxifen-treated *Ptch^+/-^NRas^fl/+^Myf5^CreER/wt^* mice. Untreated mice served as controls. Data from qRT-PCR analyses represent measurements in technical triplicates normalized to *18S rRNA* (18S) shown as fold expression of the same gene in normal muscle of the same mouse, which was set to 1. Bars show mean ± SEM and dots indicate results from individual tumors. **E, F, G)** representative immunohistochemical stainings of ERMS sections derived from the 3 genotypes using antibodies against anti-tropomyosin 3 and anti-myosin heavy chain. Please note, that in comparison to the mRNA analysis, the stainings gave no clear-cut results regarding overexpression of tropomyosin in tumors derived from tamoxifen-treated *Ptch^+/-^NRas^fl/+^Myf5^CreER/wt^* mice. Scale bar 100 µm.


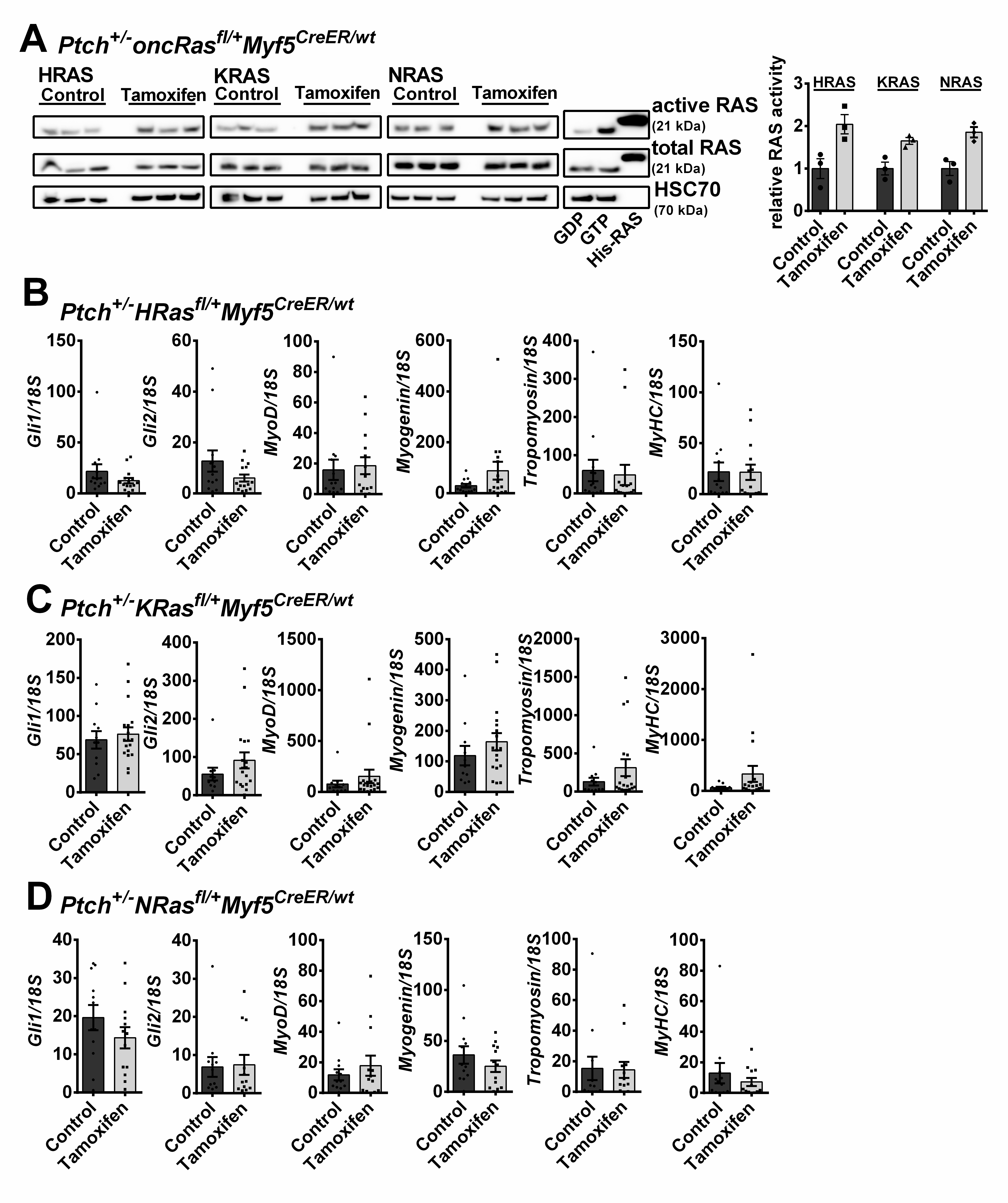


**Figure S4: Hh signaling activity or expression of muscle differentiation markers upon tamoxifen application or oncRAS induction at the full-blown ERMS stage of *Ptch^+/-^* mice, respectively.**

**A)** Representative Western blots (left panels, n=3 for each cohort) and densitometric evaluation (right graph) of active RAS in ERMS of control or tamoxifen-treated *Ptch^+/-^oncRas^fl/+^Myf5^CreER/wt^* mice. **B, C, D)** qRT-PCR analyses of the Hh signaling molecules *Gli1*, *Gli2* and the muscle markers *MyoD*, *Myogenin*, *Tropomyosin 3* and *Myosin heavy chain* (MyHC) in ERMS of (B) *Ptch^+/-^HRas^fl/+^Myf5^CreER/wt^*, (C) *Ptch^+/-^KRas^fl/+^Myf5^CreER/wt^* or (D) *Ptch^+/-^NRas^fl/+^Myf5^CreER/wt^* mice. For analysis, ERMS and skeletal muscle tissue samples were isolated from 13 control and 15 tamoxifen-treated *Ptch^+/-^HRas^fl/+^Myf5^CreER/wt^* mice, 11 control and 18 tamoxifen-treated *Ptch^+/-^KRas^fl/+^Myf5^CreER/wt^* mice and 12 control and 13 tamoxifen-treated *Ptch^+/-^NRas^fl/+^Myf5^CreER/wt^* mice.





**Figure S5: Lack of tamoxifen-induced effects on ERMS of *Ptch*^+/-^ mice**

Upper panels: ERMS growth (left graph), and mean relative growth of individual tumors (middle graph) monitored by µCT measurements before and 7 weeks after tamoxifen-treatment in *Ptch^+/-^* mice. Percentage of Ki67^+^ nuclei in the respective tumors is shown in the right graph. ERMS tissue sections from solvent-treated control (n = 29 ERMS from 17 mice) and tamoxifen-treated (n = 27 ERMS from 18 mice) *Ptch^+/-^* mice were analyzed. Lower panel: qRT-PCR analyses of the Hh signaling molecules *Gli1*, *Gli2* and the muscle markers *MyoD*, *Myogenin*, *Tropomyosin 3* and *Myosin heavy chain* (MyHC) in ERMS of *Ptch^+/-^* mice. ERMS and skeletal muscle tissue samples were isolated from 19 control and 20 tamoxifen-treated *Ptch^+/-^* mice.

Data from qRT-PCR analyses represent measurements in technical triplicates normalized to *18S rRNA* (18S) shown as fold expression of the same gene in normal muscle of the same mouse, which was set to 1. Bars show mean ± SEM. Dots indicate results from individual tumors.

**Supplementary References**

1 Schaaf G, Hamdi M, Zwijnenburg D, Lakeman A, Geerts D, Versteeg R *et al*. Silencing of SPRY1 triggers complete regression of rhabdomyosarcoma tumors carrying a mutated RAS gene. *Cancer research* 2010; 70: 762-771.

2 Martinelli S, McDowell HP, Vigne SD, Kokai G, Uccini S, Tartaglia M *et al*. RAS signaling dysregulation in human embryonal Rhabdomyosarcoma. *Genes, chromosomes & cancer* 2009; 48: 975-982.

3 Chardin P, Yeramian P, Madaule P, Tavitian A. N-ras gene activation in the RD human rhabdomyosarcoma cell line. *International journal of cancer* 1985; 35: 647-652.

4 Kang TW, Yevsa T, Woller N, Hoenicke L, Wuestefeld T, Dauch D *et al*. Senescence surveillance of pre-malignant hepatocytes limits liver cancer development. *Nature* 2011; 479: 547-551.

5 Lauth M, Bergstrom A, Shimokawa T, Tostar U, Jin Q, Fendrich V *et al*. DYRK1B-dependent autocrine-to-paracrine shift of Hedgehog signaling by mutant RAS. *Nat Struct Mol Biol* 2010; 17: 718-725.

6 Tomayko MM, Reynolds CP. Determination of subcutaneous tumor size in athymic (nude) mice. *Cancer chemotherapy and pharmacology* 1989; 24: 148-154.

7 Scholl FA, Betts DR, Niggli FK, Schafer BW. Molecular features of a human rhabdomyosarcoma cell line with spontaneous metastatic progression. *British journal of cancer* 2000; 82: 1239-1245.

8 Zibat A, Uhmann A, Nitzki F, Wijgerde M, Frommhold A, Heller T *et al*. Time-point and dosage of gene inactivation determine the tumor spectrum in conditional Ptch knockouts. *Carcinogenesis* 2009; 30: 918-926.

9 Biressi S, Bjornson CR, Carlig PM, Nishijo K, Keller C, Rando TA. Myf5 expression during fetal myogenesis defines the developmental progenitors of adult satellite cells. *Developmental biology* 2013; 379: 195-207.

10 Hahn H, Nitzki F, Schorban T, Hemmerlein B, Threadgill D, Rosemann M. Genetic mapping of a Ptch1-associated rhabdomyosarcoma susceptibility locus on mouse chromosome 2. *Genomics* 2004; 84: 853-858.

11 Nitzki F, Zibat A, Frommhold A, Schneider A, Schulz-Schaeffer W, Braun T *et al*. Uncommitted precursor cells might contribute to increased incidence of embryonal rhabdomyosarcoma in heterozygous Patched1-mutant mice. *Oncogene* 2011; 30: 4428-4436.

12 Soriano P. Generalized lacZ expression with the ROSA26 Cre reporter strain. *Nature genetics* 1999; 21: 70-71.

13 Chen X, Mitsutake N, LaPerle K, Akeno N, Zanzonico P, Longo VA *et al*. Endogenous expression of Hras(G12V) induces developmental defects and neoplasms with copy number imbalances of the oncogene. *Proceedings of the National Academy of Sciences of the United States of America* 2009; 106: 7979-7984.

14 Tuveson DA, Shaw AT, Willis NA, Silver DP, Jackson EL, Chang S *et al*. Endogenous oncogenic K-ras(G12D) stimulates proliferation and widespread neoplastic and developmental defects. *Cancer cell* 2004; 5: 375-387.

15 Haigis KM, Kendall KR, Wang Y, Cheung A, Haigis MC, Glickman JN *et al*. Differential effects of oncogenic K-Ras and N-Ras on proliferation, differentiation and tumor progression in the colon. *Nature genetics* 2008; 40: 600-608.

16 Metzger D, Chambon P. Site- and time-specific gene targeting in the mouse. *Methods* 2001; 24: 71-80.

17 Osborne DR, Yan S, Stuckey A, Pryer L, Richey T, Wall JS. Characterization of X-ray Dose in Murine Animals Using microCT, a New Low-Dose Detector and nanoDot Dosimeters. *Plos One* 2012; 7: e49936.

18 Struffert T, Doelken M, Adamek E, Schwarz M, Engelhorn T, Kloska S *et al*. Flat-detector computed tomography with intravenous contrast material application in experimental aneurysms: comparison with multislice CT and conventional angiography. *Acta Radiol* (Comparative Study) 2010; 51: 431-437.

19 Pelczar P, Zibat A, van Dop WA, Heijmans J, Bleckmann A, Gruber W *et al*. Inactivation of Patched1 in mice leads to development of gastrointestinal stromal-like tumors that express Pdgfralpha but not kit. *Gastroenterology* 2013; 144: 134-144 e136.

20 Marklein D, Graab U, Naumann I, Yan T, Ridzewski R, Nitzki F *et al*. PI3K inhibition enhances doxorubicin-induced apoptosis in sarcoma cells. *Plos One* 2012; 7: e52898.

21 Uhmann A, Dittmann K, Nitzki F, Dressel R, Koleva M, Frommhold A *et al*. The Hedgehog receptor Patched controls lymphoid lineage commitment. *Blood* 2007; 110: 1814-1823.
